# Synthesis, fungicidal evaluation and 3D-QSAR studies of novel 1,3,4-thiadiazole xylofuranose derivatives

**DOI:** 10.1371/journal.pone.0181646

**Published:** 2017-07-26

**Authors:** Guanghui Zong, Xiaojing Yan, Jiawei Bi, Rui Jiang, Yinan Qin, Huizhu Yuan, Huizhe Lu, Yanhong Dong, Shuhui Jin, Jianjun Zhang

**Affiliations:** 1 Key Laboratory of Pesticide Chemistry and Application Technology, College of Science, China Agricultural University, Beijing, China; 2 The Institute of Plant Protection, Chinese Academy of Agricultural Sciences, Beijing, China; National Cancer Institute at Frederick, UNITED STATES

## Abstract

1,3,4-Thiadiazole and sugar-derived molecules have proven to be promising agrochemicals with growth promoting, insecticidal and fungicidal activities. In the research field of agricultural fungicide, applying union of active group we synthesized a new set of 1,3,4-thiadiazole xylofuranose derivatives and all of the compounds were characterized by ^1^H NMR and HRMS. In precise toxicity measurement, some of compounds exhibited more potent fungicidal activities than the most widely used commercial fungicide Chlorothalonil, promoting further research and development. Based on our experimental data, 3D-QSAR (three-dimensional quantitative structure-activity relationship) was established and investigated using comparative molecular field analysis (CoMFA) and comparative molecular similarity indices analysis (CoMSIA) techniques, helping to better understand the structural requirements of lead compounds with high fungicidal activity and environmental compatibility.

## Introduction

1,3,4-Thiadiazole is a privileged five-membered heterocyclic scaffold with interesting properties, incorporation which often improves the desirable properties of the active molecules in medicinal chemistry. [1–6] Besides being used as drugs, 1,3,4-thiadiazole and their derivatives have also been widely applied as agrochemicals with a broad spectra of bioactivities, [7–13] among which their fungicidal activity particularly attracted our attention as part of the comprehensive project for developing agricultural fungicides in our group. [14–17]

Sugar-derived molecules participate in various vital processes, exhibiting crucial physiological and biological activities, especially in specific molecular recognition. Many natural products composed of carbohydrate moieties show great bioactivities, which make them widely used as drugs and pesticides. [18, 19] Besides the bioactivities, carbohydrates have also been widely used to modify small biomolecules to tune their physical properties, such as water solubility and p*K*_*a*_ values to increase the bioactivities and/or decrease toxicities. [20]

**Fig 1:**
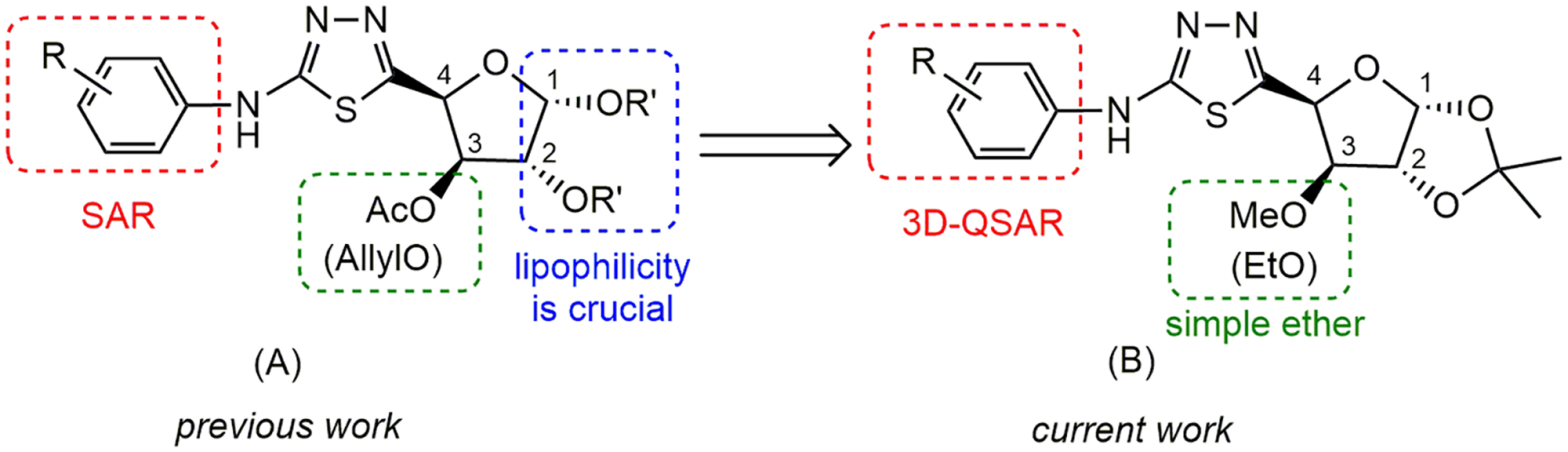
Design strategy for target compounds.

With the idea of utilising the unique bioactivities of sugar-derived molecules, we have reported a hybrid of D-xylofuranose and 1,3,4-thiadiazole with promising fungicidal properties and found that the lipophilicity of these compounds is one of the key parameters for their fungicidal activities (Fig 1A). [17] As a continuation in our endeavour of searching for more effective fungicidal agents, we have designed a new series of structures (Fig 1B) containing 1,2-*O*-isopropylidene to retain the lipophilicity, and replaced the 3-*O*-moieties with simple ethers. Twenty-two new xylofuranose-1,3,4-thiadiazole derivatives were synthesized and bioassayed. Furthermore, we have studied the COMFA and CoMSIA models through researching structure-activity relationship, which may be used in designing and predicting the fungicidal activity of novel molecules.

## Results and discussion

### Synthesis

**Fig 2:**
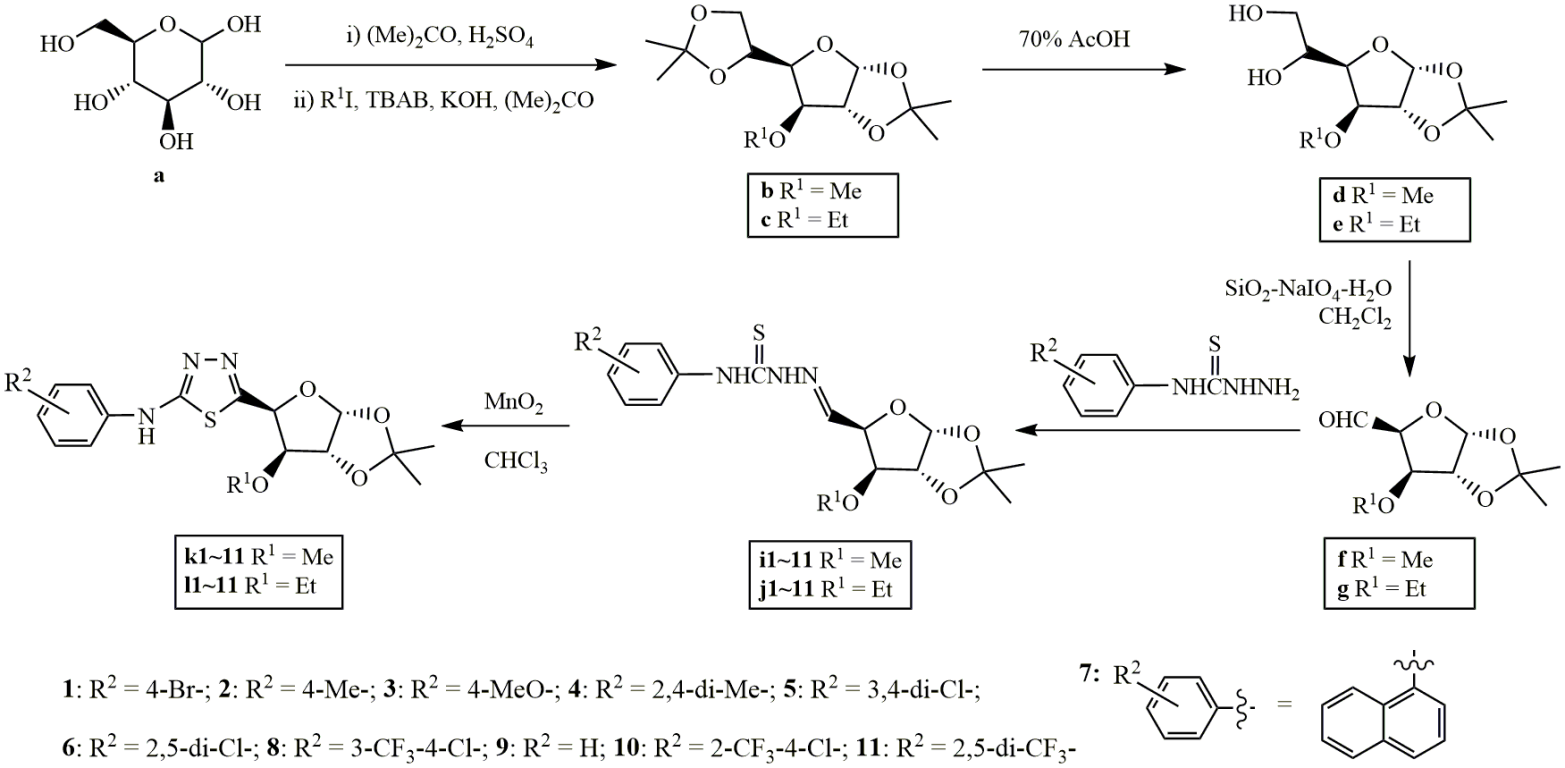
Synthesis of the title compounds k/l.

**Fig 3:**
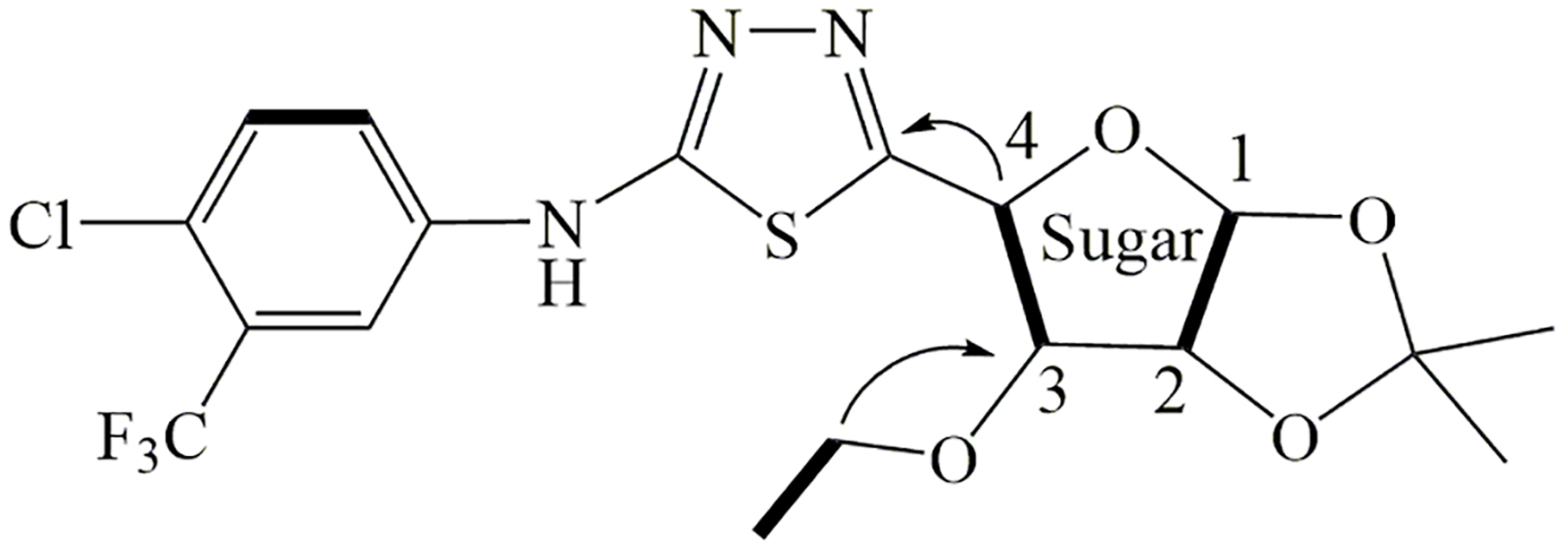
Key COSY (bold) and HMBC (arrows) correlations in l8.

Synthesis of the title compounds was achieved by coupling 3-*O*-substituted furanosyl aldehydes (**f** and **g**) and substituted thiosemicarbazides (**h**) in refluxing CH_2_Cl_2_, followed by oxidative cyclization over MnO_2_ with an overall yield of 68%–91% over two steps (Fig 2). The proton (on chiral carbons) assignments for title compounds were done with the aid of 2D NMRs, including COSY, HSQC and HMBC NMRs. The typical COSY and HMBC correlations in a representative compound **l8** are illustrated in Fig 3. The two key intermediates, i.e., aldehydes (**f** and **g**) and thiosemicarbazides (**h**), were obtained from commercially available D-glucose and substituted arylamines as starting materials following the literature reported procedure. [21–23]

### Preliminary measurement of fungicidal activity

In vitro fungicidal activities of title compounds **k**/**l** against six fungal species (S. Sclerotiorum, P. CapasiciLeonian, B. Cinerea, R. Solani, P. Oryae and P. asparagi) were first tested at a concentration of 50 μg/mL (see S1 Table). The bioassay results showed that the title compounds exhibited significant fungicidal activities against the six tested species, especially against Sclerotinia sclerotiorum.Thirteen out of the twenty-two tested compoundsshowed 90% or more inhibition against S. sclerotiorum at this concentration. However, the number of the tested title compounds with an inhibition rate over 90% against P. CapasiciLeonian, Botrytis cinerea, Rhizoctonia solani, Pyricularia oryae and Phomopsis asparagi was 5, 6, 0, 4 and 4, respectively. Compounds **k1**, **k5**, **k6**, **k8**, **l5**, **l6** and **l8** are the most broad-spectrum boasting inhibitition rates over 90% for at least three tested fungi.

### Precise toxicity measurement of fungicidal activity

Since most of the title compounds exhibited excellent fungicidal activity against S. sclerotiorum, precise bioassay against this fungi was carried out. As shown in Table 1, more than half of the title compounds (13/22) showed promising fungicidal activity against S. Sclerotiorum with EC_50_ values lower than 3 μg/mL. Particularly, compounds **k1**, **k8**, **l1** and **l5** (the EC_50_ values of which are 0.52, 0.43, 0.46 and 0.57 μg/mL, respectively) showed comparable fungicidal activity with the commercial fungicide chlorothalonil (EC_50_ = 0.59 μg/mL).

**Table 1:** EC_50_ and EC_90_ values of target compounds against S. sclerotiorum.

Compd.	toxic regression equation	EC_50_	EC_90_	correlation coefficient R
**k1**	Y = 5.24+0.85x	0.52	16.78	0.9377
**k2**	Y = 5.08+0.62x	0.75	86.27	0.9266
**k3**	Y = 4.24+0.87x	7.51	221.00	0.9772
**k4**	Y = 3.48+1.44x	11.42	89.25	0.9654
**k5**	Y = 4.87+1.17x	1.29	16.04	0.9798
**k6**	Y = 4.06+1.48x	4.34	32.03	0.9471
**k7**	Y = 5.00+0.93x	0.99	23.50	0.9691
**k8**	Y = 5.36+1.00x	0.43	8.40	0.9673
**k9**	Y = 3.71+1.23x	11.07	121.16	0.9347
**k10**	Y = 3.26+1.86x	8.55	41.71	0.9332
**k11**	Y = 4.73+0.73x	2.35	135.00	0.9488
**l1**	Y = 5.26+0.79x	0.46	19.42	0.8998
**l2**	Y = 4.85+1.04x	1.39	23.99	0.9375
**l3**	Y = 4.19+1.18x	4.83	58.36	0.9979
**l4**	Y = 4.56+1.18x	2.35	28.96	0.9506
**l5**	Y = 5.24+0.99x	0.57	11.25	0.9729
**l6**	Y = 4.56+1.34x	2.12	19.15	0.9719
**l7**	Y = 3.83+1.73x	4.78	26.37	0.9955
**l8**	Y = 5.21+1.14x	0.66	8.73	0.9993
**l9**	Y = 3.08+1.79x	11.69	60.51	0.9573
**l10**	Y = 3.63+1.86x	5.43	26.55	0.9996
**l11**	Y = 4.72+1.00x	1.92	36.40	0.9723
**Chlorothalonil**	Y = 5.19+0.84x	0.59	19.56	0.9784

Prediction of the LogP rate of target compound and toxicity is presented in Supporting Information (S3 Table).

### CoMFA and CoMSIA model

**Fig 4:**
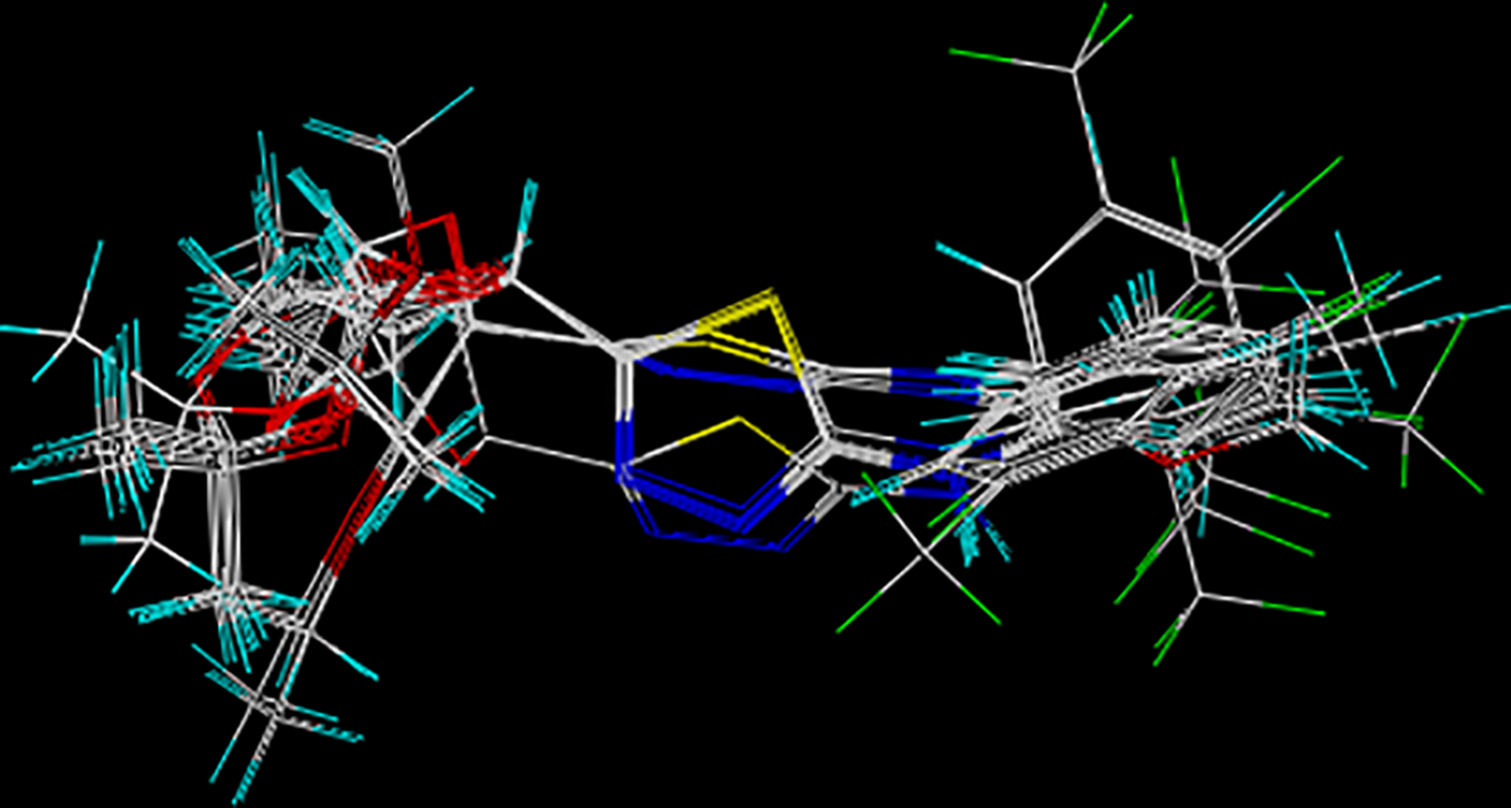
Image of superimposed structures.

In the target molecules, there are two variable groups which are substituent R^1^ on the sugar ring and R^2^ on the benzene ring. Comparative molecular field analysis (CoMFA) and comparative molecular similarity indices analysis (CoMSIA) were applied to research the relationship of substituents and inhibitory activity. The result of molecular superimposition is shown in Fig 4.

As shown in Table 2, all selected compounds in the training set were aligned with each other based on the template **k8**. The CoMFA model exhibited contribution of steric (59.5%) and electrostatic (40.5%) fields. The cross-validation coefficient $r_{\text{cv}}^{2}$ was given with 0.639 in the obtained CoMFA model, which was greater than 0.5 and indicated a good significance. An optimum number of components of 5 and a non-cross-validated r^2^_ncv_ of 0.949 were observed with this model. The high F value (67.036) suggests that the model is meaningful.

**Table 2:** COMFA and COMSIA analysis results*.

Parameter	COMFA	COMSIA Model1	COMSIA Model2	COMSIA Model3	COMSIA Model4
*q*^2^	0.639	0.528	0.508	0.486	0.495
*r*^2^	0.968	0.964	0.962	0.913	0.945
SE	0.110	0.116	0.120	0.174	0.144
F	67.036	59.371	55.513	31.587	37.969
Components relative field contributions(%)	5	5	5	4	5
S	59.5	11.9	11.5	-	-
E	40.5	35.3	33.3	40.2	48.8
H	-	37.0	35.5	44.3	51.2
D	-	15.9	14.6	15.5	-
A	-	-	5.1	-	-

*Model 1: S+E+H+D; Model 2: S+E+H+D+A; Model 3: E+H+D; Model 4: E+H.

Training set: **k1**, **k3**, **k4**, **k5**, **k6**, **k8**, **k9**, **k10**, **l1**, **l2**, **l3**, **l5**, **l6**, **l7**, **l8**, **l10**, **l11**.

Test set: **k2**, **k7**, **k11**, **l4**, **l9**.

To investigate the significance of hydrophobic and H-bond fields on the activities, CoMSIA analysis was performed using steric, electrostatic, hydrophobic, and H bond donor and acceptor descriptors. Considering the combination of all the fields, the results are displayed in Table 2. As shown in the table, the combination of steric field, electrostatic field, hydrophobic field, and hydrogen bond acceptor field was proven to be the best model with r^2^_cv_ 0.528 at five components, r^2^_ncv_ 0.964.

**Fig 5:**
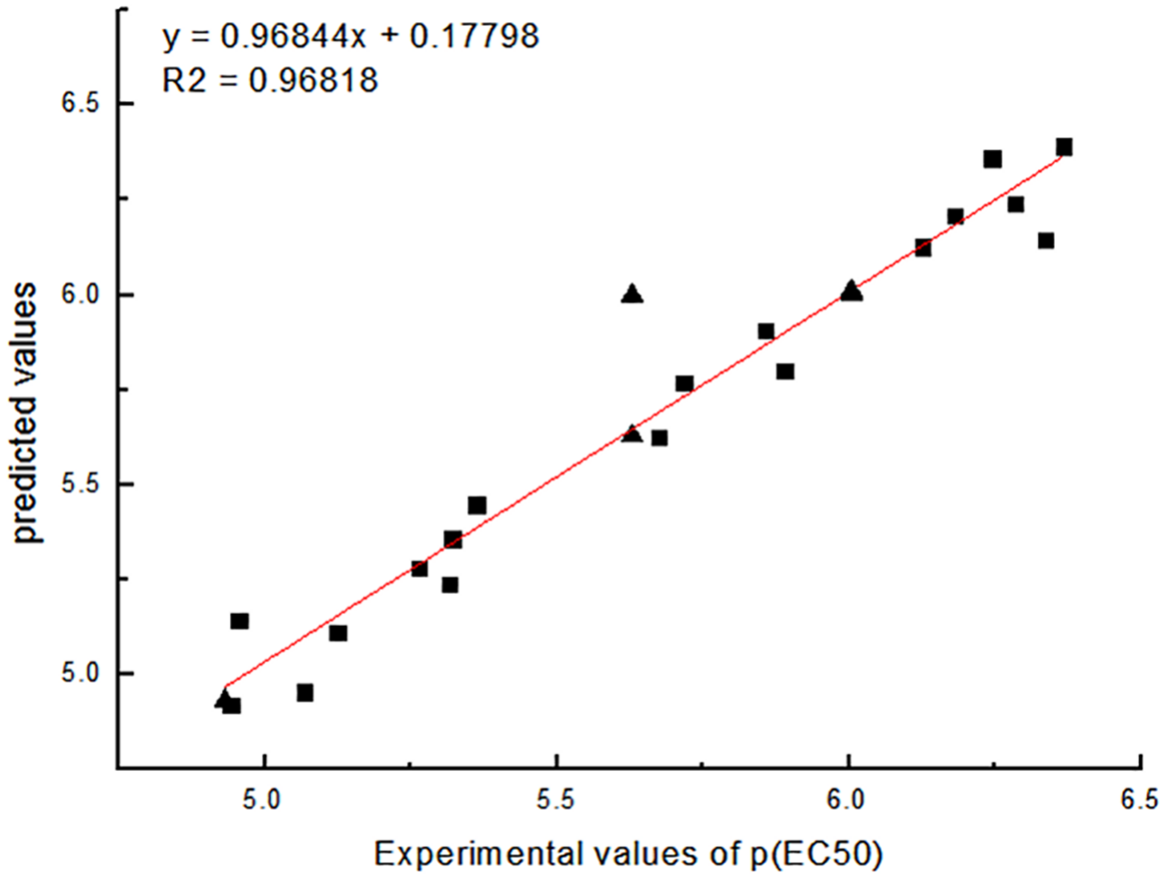
The correlation between the experimental values and predicted of COMFA (training set ■, test set ▲).

**Fig 6:**
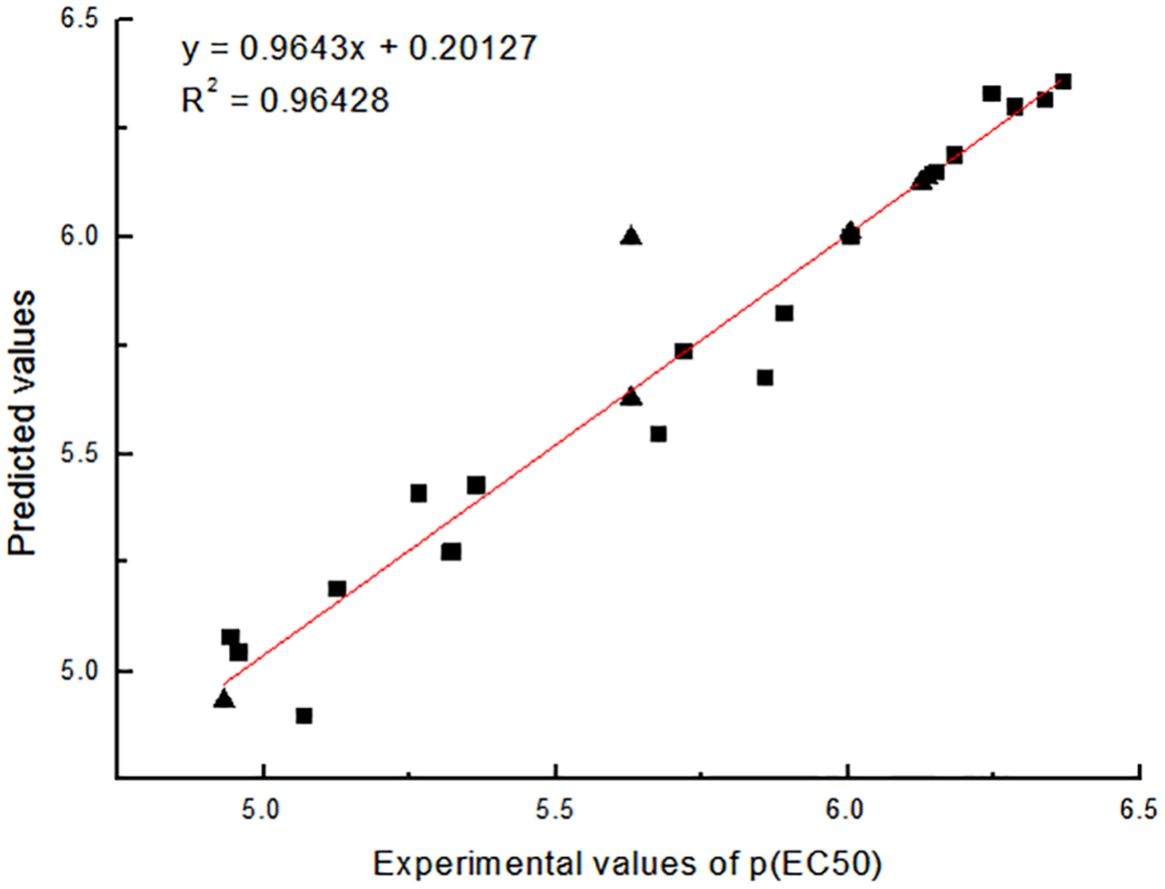
The correlation between the experimental values and predicted of COMSIA (training set ■, test set ▲).

The graph depicting the calculated vs observed activities of training and test set molecules are shown in Figs 5 and 6, respectively. The correlation coefficient of 0.96818 and 0.96428 for CoMFA and CoMSIA model, respectively, further supported the significance of the selected models.

The Coefficient of cross validation q^2^ of COMFA and COMSIA models are greater than 0.5, so the established 3D-QSAR model has good prediction ability. Table 3 shows the relationship of the predicted values and experimental values.

**Table 3:** The experimental value, forecast and difference of CoMFA and CoMSIA models.

Compd.	Experimental values of p(EC_50_)	CoMFA		CoMSIA Model 1	
		predicted values	D-value	predicted values	D-value
**k1**	6.284	6.24	0.044	6.301	-0.017
**k2**	6.1249	6.125	0	6.125	0
**k3**	5.1244	5.111	0.013	5.192	-0.068
**k4**	4.9423	4.922	0.02	5.079	-0.137
**k5**	5.8894	5.8	0.089	5.827	0.062
**k6**	5.3625	5.449	-0.087	5.429	-0.067
**k7**	6.0044	6.004	0	6.004	0
**k8**	6.3665	6.39	-0.023	6.359	0.007
**k9**	4.9559	5.143	-0.187	5.046	-0.09
**k10**	5.068	4.956	0.112	4.9	0.168
**k11**	5.6289	5.998	-0.369	5.998	-0.369
**l1**	6.3372	6.144	0.193	6.318	0.019
**l2**	5.857	5.906	-0.049	5.679	0.178
**l3**	5.3161	5.239	0.077	5.274	0.042
**l4**	5.6289	5.629	0	5.629	0
**l5**	6.2441	6.36	-0.115	6.331	-0.087
**l6**	5.6737	5.625	0.049	5.549	0.124
**l7**	5.3206	5.359	-0.038	5.278	0.042
**l8**	6.1805	6.207	-0.027	6.19	-0.01
**l9**	4.9322	4.932	0	4.932	0
**l10**	5.2652	5.283	-0.018	5.412	-0.147
**l11**	5.7167	5.769	-0.053	5.738	-0.021

### 3D-QSAR contour maps

**Fig 7:**
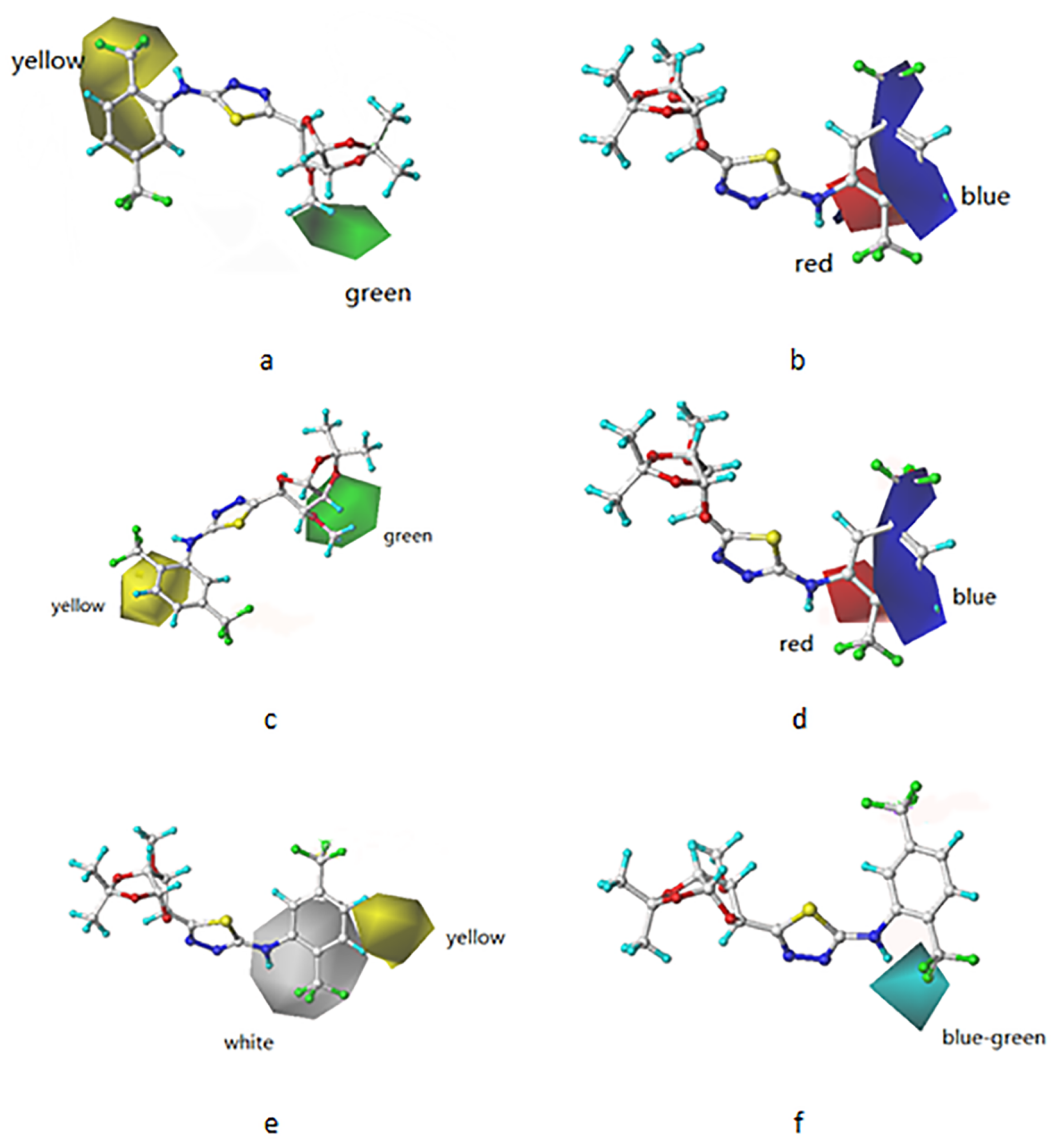
Contour plots (a) CoMSIA Steric. (b) CoMFA Electrostatic. (c) CoMSIA Steric. (d) CoMSIA Electrostatic. (e) CoMSIA Hydrophobic. (f) CoMSIA Hydrogen bond receptor. Compound k8 in cap and stick is shown.

The steric and electrostatic contour maps of the COMFA and COMSIA models are shown in Fig 7a and 7b. Compound **k8** was used as the reference structure. Sterically favored areas (contribution level 80%) were represented by green polyhedral while sterically disfavored areas (contribution level 20%) were represented by yellow polyhedral. Furthermore, the blue and red contours (80 and 20% contributions) depicted the positions where positively charged groups and negatively charged groups would be favorable, respectively. The sterically favored green contour could be found around R^2^ which indicated that increasing bulky groups at R^2^ position were advantageous for activity while R^1^ remained the same, e.g., EC_50_(**k1**) > EC_50_(**l1**), EC_50_(**k3**) > EC_50_(**l3**), EC_50_(**k4**) > EC_50_(**l4**), EC_50_(**k5**) > EC_50_(**l5**), EC_50_(**k6**) > EC_50_(**l6**), EC_50_(**k10**) > EC_50_(**l10**), EC_50_(**k11**) > EC_50_(**l11**). A large yellow region overlapping R^2^, which was coincident with our CoMFA result, verified that a smaller R^2^ group was an essential factor for activity. For **k2** and **l4**, the presence of a methyl group on the benzene ring of R^2^ decreased p(EC_50_) from 6.12 to 4.94. In Fig 7b, the blue polyhedral covering the meta-position of benzene ring indicated that the presence of electron-rich groups could not enhance the biological activity.

The COMSIA model contour maps, derived using steric, electrostatic, hydrophobic and hydrogen bond acceptor fields, are shown in Fig 7c–7f. Compound **k8** was used as the reference molecule. Fig 7c and 7d, which were more or less similar to Fig 7a and 7b, represented steric and electrostatic contour maps, respectively. In Fig 7e the yellow contours represented regions where hydrophobic substituents would increase the activity, while the white contours represented regions where the hydrophobic group would be unfavorable. The ortho-position of R^2^ was covered by a white region. Take compounds **k6**/**l6** as an example. A Cl atom is in the area. Because of the good hydrophilicity of Cl, the activity was increased. There was a yellow region at the meta-position of R^2^. Take compounds **k7**/**l7** and **k9**/**l9** as an example. It was clear that compounds with naphthyl (**k7**/**l7**) showed higher activity than compounds with less hydrophobic benzene ring (**k9**/**l9**). In Fig 7d, the magenta and red contours depicted the position where hydrophobic groups would be favorable or unfavorable, respectively. From the Fig 7f, we can conclude that if hydrogen bond acceptors was added at the ortho-position of benzene ring, the activity will improve.

To identify the putative targets, the structure information of the title compounds has been submited to Pharm Mapper Server [http://lilab.ecust.edu.cn/pharmmapper/index.php] [24, 25] and the resulting targets prediction of the 22 compounds and the highest fit score are shown in Table 4. In addition, the targets prediction and the normalized fit score are shown in Table 5. According to the normalized fit score, the Carbonic anhydrase 2(PDB ID: 1ZGF) is the most suitable target for our compounds. The feature number and the target prediction are shown in supporting information (S4 Table).

**Table 4:** The target name, the PDB ID and fit score of 22 compounds.

Compd.	PDB ID	Target Name	Fit Score
**k1**	2CEK	Acetylcholinesterase	6.575
**k2**	1TCX	Gag-Pol polyprotein	7.332
**k3**	1LWL	Camphor 5-monooxygenase	6.511
**k4**	1LN3	Phosphatidylcholine transfer protein	7.411
**k5**	2R43	Gag-Pol polyprotein	7.044
**k6**	1LN3	Phosphatidylcholine transfer protein	7.143
**k7**	3FNH	Enoyl-[acyl-carrier-protein] reductase [NADH]	6.916
**k8**	3DCT	Bile acid receptor	7.8
**k9**	1LWL	Camphor 5-monooxygenase	6.477
**k10**	1LN3	Phosphatidylcholine transfer protein	7.093
**k11**	3DCT	Bile acid receptor	7.8
**l1**	1TCX	Gag-Pol polyprotein	7.371
**l2**	3DCU	Bile acid receptor	7.298
**l3**	1MEU	Gag-Pol polyprotein	7.384
**l4**	1LN3	Phosphatidylcholine transfer protein	7.518
**l5**	2CEK	Acetylcholinesterase	7.24
**l6**	1MEU	Gag-Pol polyprotein	7.835
**l7**	1TCX	Gag-Pol polyprotein	7.511
**l8**	2CEK	Acetylcholinesterase	7.208
**l9**	1MEU	Gag-Pol polyprotein	6.798
**l10**	1G2N	NONE	7.338
**l11**	1G2N	NONE	8.602

**Table 5:** The target name, the PDB ID and normalized fit score of 22 compounds.

Compd.	PDB ID	Target Name	Normalized Fit Score
**k1**	1ZGF	Carbonic anhydrase 2	0.79
**k2**	1ZGF	Carbonic anhydrase 2	0.856
**k3**	1ZGF	Carbonic anhydrase 2	0.7274
**k4**	1ZGF	Carbonic anhydrase 2	0.8581
**k5**	1ZGF	Carbonic anhydrase 2	0.8229
**k6**	1G48	Carbonic anhydrase 2	0.8733
**k7**	1IF8	Carbonic anhydrase 2	0.8492
**k8**	1F4F	Thymidylate synthase	0.7001
**k9**	1ZGF	Carbonic anhydrase 2	0.7231
**k10**	1ZGF	Carbonic anhydrase 2	0.8161
**k11**	1F4F	Thymidylate synthase	0.7001
**l1**	1BN4	Carbonic anhydrase 2	0.8544
**l2**	1ZGF	Carbonic anhydrase 2	0.8562
**l3**	1I8Z	Carbonic anhydrase 2	0.8473
**l4**	1ZGF	Carbonic anhydrase 2	0.8551
**l5**	1ZGF	Carbonic anhydrase 2	0.8229
**l6**	1G48	Carbonic anhydrase 2	0.877
**l7**	1IF8	Carbonic anhydrase 2	0.8658
**l8**	1BN4	Carbonic anhydrase 2	0.8351
**l9**	1ZGF	Carbonic anhydrase 2	0.7259
**l10**	1ZGF	Carbonic anhydrase 2	0.8185
**l11**	1G48	Carbonic anhydrase 2	0.8677

Compared to our previous study, [17] twenty-two new 1,3,4-thiadiazole xylofuranose derivatives with different moiety on C-3 of sugar ring were synthesized and bioassayed in the present work. Based on the structure and fungicidal activity results, 3D-QSAR was established and investigated using CoMFA and CoMSIA. The established models will facilitate the development of more potent pesticide molecules.

## Methods and materials

### General methods

All starting materials and reagents were commercially available and used without further purification except as indicated. ^1^H-NMR (300 MHz) and ^13^C-NMR (75 MHz) spectra were recorded in CDCl_3_ or DMSO-d6 with a Bruker DPX300 spectrometer, using TMS as internal standard; Mass spectra were obtained with Agilent 1100 series LC/MSD mass spectrometer. High-resolution mass spectra (HRMS) was performed by Peking University. Melting points were measured on a Yanagimoto melting-point apparatus and are uncorrected.

Chemical synthesis.

General procedure for the syntheses of substituted aldehydes **f** and **g**. [22]

Compound **a** (26 g, 0.10 mol) was dissolved in anhydrous acetone (150mL) containing potassium hydroxide (7.4 g, 0.13 mol) and tetrabutyl ammonium bromide (1.2 g, 3.7mmol), then iodomethane (7.6 mL, 0.15 mol) was added dropwise to the solution over 30 min at –10°C. The temperature was slowly raised to r.t. and the mixture was stirred for another 1 h; TLC (PE–EtOAc, 3:1) indicated completion. The solution was concentrated and then the mixture was diluted with CH_2_Cl_2_ (100 mL), washed with water (3 ×100 mL), and dried (Na_2_SO_4_). The solution was concentrated and the crude product b/c could be directly used for the next step without further separation and purification.

Compound **b**/**c** (0.1 mol) was dissolved in 70% AcOH (200 mL) and stirred for 1.5 h at 75°C; TLC (PE–EtOAc, 2:1) indicated completion. The mixture was concentrated under reduced pressure and then co-evaporated with toluene (3 × 100 mL). The crude product **d**/**e** was obtained and could be directly used for the next step without further separation and purification.

To a stirred solution of SiO_2_-NaIO_4_-H_2_O (100g) in CH_2_Cl_2_ (200 mL) was added Compound **d**/**e** (0.1 mol) in CH_2_Cl_2_ over 30 min at r.t. The mixture was stirred for another 1 h; TLC (PE–EtOAc, 3:1) indicated completion. The mixture was filtered and the the solution was concentrated, and purification of the residue by column chromatography (silica gel, PE–EtOAc, 4:1) gave **g**/**f** as a white solid in 87% overall yields. General procedure for the syntheses of oxidation system SiO_2_-NaIO_4_-H_2_O.

To a 70°C solution of NaIO_4_ (25.7 g, 0.12 mol) in deionized water (100 mL) was added 200-300 mesh silica gel (100g) in several portions, and the system was stirred for 0.5 h. The oxidation system SiO_2_-NaIO_4_-H_2_O was obtained and could be used for the reactions directly. General procedure for the synthesis of intermediate compounds **i**/**j**.

A solution of aldehyde **f**/**g** (5.5 mmol) and thiosemicarbazide **h** (5 mmol) in CH_2_Cl_2_ (100 mL) was heated to reflux for 6 h, at the end of which time TLC (eluent: 2:1 petroleum ether-EtOAc) indicated that the reaction was complete. The solvent was evaporated under diminished pressure at 40°C to give a white solid, and the crude product was used for next step directly without purification. General procedure for the synthesis of title compounds **k**/**l**.

To a stirred solution of compound **i**/**j** (5.0 mmol) in CHCl_3_ (80 mL) was added MnO_2_ (10 g). The mixture was stirred for a further 1 h, at the end of which time TLC (eluent: 2:1 petroleum ether-EtOAc) indicated that the reaction was complete. After filtration, the filtrate was evaporated under reduced pressure to give a crude product, which was purified on silica gel column chromatography with 4:1 petroleum ether-EtOAc as the eluent to give the compounds **k**/**l**.

2-(4-Bromophenylamino)-5-(2R,3S-O-isopropylidene-4S-O-methyl-tetrahydrofuro-2,3,4-triol-5S)-1,3,4-thiadiazol (**k1**) Yield: 89%. White solid, mp 217.8-218.3°C. ^1^H-NMR (CDCl_3_):*δ* 10.49 (s, 1H, NH), 7.50-7.47 (m, 2H, ArH), 7.37–7.34 (m, 2H, ArH), 6.01 (d, *J* = 3.7 Hz, 1H,H-1), 5.61 (d, *J* = 3.1 Hz, 1H, H-4), 4.71 (d, *J* = 3.7 Hz, 1H, H-2), 3.96 (d, *J* = 3.1 Hz, 1H, H-3), 3.33 (s, 3H, OCH_3_), 1.58, 1.35 (2s, 6H, Me_2_C). ESI-MS m/z for C_16_H_18_BrN_3_O_4_S [M-H] Found: 425.9. HRMS calcd. for C_16_H_18_BrN_3_O_4_S [M+H]+ 428.02742. Found: 428.02686

2-(4-Tolylamino)-5-(2R,3S-O-isopropylidene-4S-O-methyl-tetrahydrofuro-2,3,4-triol-5S)-1,3,4-thiadiazole (**k2**) Yield: 75%. Pale yellow solid, mp 172.8-173.7°C. ^1^H-NMR (CDCl_3_):*δ* 10.05(s, 1H, NH), 7.33-7.16 (m, 4H, ArH), 6.00 (d, *J* = 3.7 Hz, 1H, H-1), 5.60 (d, *J* = 3.2 Hz, 1H, H-4), 4.69 (d, *J* = 3.7 Hz, 1H, H-2), 3.96 (d, *J* = 3.1 Hz, 1H, H-3), 3.30 (s, 3H, OCH_3_), 2.33(s, 3H, Ar-CH_3_), 1.57, 1.36 (2s, 6H, Me_2_C). ESI-MS m/z for C_17_H_21_N_3_O_4_S [M+H]+ Found: 364.0. HRMS calcd. for C_17_H_21_N_3_O_4_S [M+H]+ 364.13255. Found: 364.13193

2-(4-Methoxyphenylamino)-5-(2R,3S-O-isopropylidene-4S-O-methyl-tetrahydrofuro-2,3,4-triol-5S)-1,3,4-thiadiazole (**k3**) Yield: 79%. White solid, mp 211.3-211.7°C. ^1^H-NMR (CDCl_3_):*δ* 10.15 (s, 1H, NH), 7.35 (d, *J* = 8.9 Hz, 2H, ArH), 6.91 (d, *J* = 8.9 Hz, 2H, ArH), 5.99 (d, *J* = 3.7 Hz, 1H, H-1), 5.59 (d, *J* = 3.1 Hz, 1H, H-4), 4.68 (d, *J* = 3.7 Hz, 1H, H-2), 3.95 (d, *J* = 3.1 Hz,1H, H-3), 3.81 (s, 3H, Ar-OCH_3_), 3.31 (s, 3H, OCH_3_), 1.56, 1.36 (2s, 6H, Me_2_C). ESI-MS m/z for C_17_H_21_N_3_O_5_S [M+Na]+ Found: 402.1. HRMS calcd. for C_17_H_21_N_3_O_5_S [M+H]+ 380.12747. Found: 380.12708

2-(2,4-Dimethylphenylamino)-5-(2R,3S-O-isopropylidene-4S-O-methyl-tetrahydrofuro-2,3,4-triol-5S)-1,3,4-thiadiazole (**k4**) Yield: 81%. Pale yellow solid, mp 107.7-107.9°C.^1^H-NMR (CDCl_3_):*δ* 7.32 (d, *J* = 8.0 Hz, 1H, ArH), 7.06–7.01 (m, 2H, ArH), 5.95 (d, *J* = 3.7 Hz, 1H, H-1), 5.52 (d, *J* = 3.1 Hz, 1H, H-4), 4.81 (s, 1H, NH), 4.65 (d, *J* = 3.7 Hz, 1H, H-2), 3.92 (d, *J* = 3.1 Hz, 1H, H-3), 3.29 (s, 3H, OCH_3_), 2.32 (2s, 6H, Ar-OCH_3_), 1.54, 1.34 (2s, 6H, Me_2_C). ESI-MS m/z for C_18_H_23_N_3_O_4_S [M+H]+ Found: 378.1. HRMS calcd. for C_18_H_23_N_3_O_4_S [M+H]+ 378.14820. Found: 378.14798

2-(3,4-Dichlorophenylamino)-5-(2R,3S-O-isopropylidene-4S-O-methyl-tetrahydrofuro-2,3,4-triol-5S)-1,3,4-thiadiazole (**k5**) Yield: 78%. White solid, mp 191.1-192.1°C.^1^H-NMR (CDCl_3_):*δ* 11.05 (s, 1H, NH), 7.59 (d, *J* = 2.6 Hz, 1H, ArH), 7.42 (d, *J* = 8.7 Hz, 1H, ArH), 7.33 (dd, *J* = 8.8, 2.7 Hz, 1H, ArH), 6.03 (d, *J* = 3.6 Hz, 1H, H-1), 5.63 (d, *J* = 3.1 Hz, 1H, H-4), 4.72 (d, *J* = 3.6 Hz, 1H, H-2), 4.00 (d, *J* = 3.2 Hz, 1H, H-3), 3.34 (s, 3H, OCH_3_), 1.59, 1.38 (2s, 6H, Me_2_C). ESI-MS m/z for C_16_H_17_Cl_2_N_3_O_4_S [M+H]+ Found: 418.0. HRMS calcd. for C_16_H_17_Cl_2_N_3_O_4_S [M+H]+ 418.03896. Found: 418.03897

2-(2,5-Dichlorophenylamino)-5-(2R,3S-O-isopropylidene-4S-O-methyl-tetrahydrofuro-2,3,4-triol-5S)-1,3,4-thiadiazole (**k6**) Yield: 91%. White solid, mp 113.2-113.4°C.^1^H-NMR (CDCl_3_):*δ* 8.21 (d, *J* = 2.3 Hz, 1H, ArH), 7.30 (d, *J* = 8.5 Hz, 1H, ArH), 7.17 (s, 1H, NH), 6.98 (dd, *J* = 8.5, 2.4 Hz, 1H, ArH), 6.01 (d, *J* = 3.6 Hz, 1H, H-1), 5.60 (d, *J* = 3.2 Hz, 1H, H-4), 4.71 (d, *J* = 3.6 Hz, 1H, H-2), 3.99 (d, *J* = 3.2 Hz, 1H, H-3), 3.32 (s, 3H, OCH_3_), 1.55, 1.36 (2s, 6H, Me_2_C). ESI-MS m/z for C_16_H_17_Cl_2_N_3_O_4_S [M+Na]+ Found: 440.0. HRMS calcd. for C_16_H_17_Cl_2_N_3_O_4_S [M+H]+ 418.03896. Found: 418.03854

2-(Naphthalen-1-ylamino)-5-(2R,3S-O-isopropylidene-4S-O-methyl-tetrahydrofuro-2,3,4-triol-5S)-1,3,4-thiadiazole (**k7**) Yield: 73%. Pale yellow solid, mp 91.0-91.8°C.^1^H-NMR (DMSO-d6):*δ* 11.18 (br-s, 1H, NH), 8.24 (m, 1H, ArH), 8.11 (d, *J* = 7.5 Hz, 1H, ArH), 7.95 (m, 1H, ArH), 7.71 (d, *J* = 8.2 Hz, 1H, ArH), 7.61-7.50 (m, 3H, ArH), 5.96 (d, *J* = 3.7 Hz, 1H, H-1), 5.35 (d, *J* = 3.1 Hz, 1H, H-4), 4.82 (d, *J* = 3.7 Hz, 1H, H-2), 3.98 (d, *J* = 3.1 Hz, 1H, H-3), 3.29 (s, 3H, OCH_3_), 1.48, 1.30 (2s, 6H, Me_2_C). ESI-MS m/z for C_20_H_21_N_3_O_4_S [M-H] Found: 398.1. HRMS calcd. for C_20_H_21_N_3_O_4_S [M+H]+ 400.13255. Found: 400.13208

2-(4-Chloro-3-(trifluoromethyl)phenylamino)-5-(2R,3S-O-isopropylidene-4S-O-methyl-tetrahydrofuro-2,3,4-triol-5S)-1,3,4-thiadiazole (**k8**) Yield: 84%. White solid, mp 140.2-140.5°C.^1^H-NMR (CDCl_3_):*δ* 11.18 (s, 1H, NH), 7.85 (d, *J* = 2.8 Hz, 1H, ArH), 7.61 (dd, *J* = 8.7, 2.7 Hz, 1H, ArH), 7.50 (d, *J* = 8.7 Hz, 1H, ArH), 6.04 (d, *J* = 3.6Hz, 1H, H-1), 5.63 (d, *J* = 3.2 Hz, 1H, H-4), 4.73 (d, *J* = 3.7 Hz, 1H, H-2), 4.01 (d, *J* = 3.1 Hz, 1H, H-3), 3.34 (s, 3H, OCH_3_), 1.59, 1.39 (2s, 6H, Me_2_C). ESI-MS m/z for C_17_H_17_ClF_3_N_3_O_4_S [M-H] Found: 449.9. HRMS calcd. for C_17_H_17_ClF_3_N_3_O_4_S [M+H]+ 452.06532. Found: 452.06512

2-(Phenylamino)-5-(2R,3S-O-isopropylidene-4S-O-methyl-tetrahydrofuro-2,3,4-triol-5S)-1,3,4-thiadiazole (**k9**) Yield: 85%. White solid, mp 174.4-174.8°C.^1^H-NMR (CDCl_3_):*δ* 10.43 (s, 1H, NH), 7.45–7.35 (m, 4H, ArH), 7.07 (m, 1H, ArH), 6.01 (d, *J* = 3.6 Hz, 1H, H-1), 5.63 (d, *J* = 3.1 Hz, 1H, H-4), 4.71 (d, *J* = 3.7 Hz, 1H, H-2), 3.98 (d, *J* = 3.1 Hz, 1H, H-3), 3.32 (s, 3H, OCH_3_), 1.58, 1.36 (2s, 6H, Me_2_C). ESI-MS m/z for C_16_H_19_N_3_O_4_S [M+Na]+ Found: 372.0. HRMS calcd. for C_16_H_19_N_3_O_4_S [M+H]+ 350.11690. Found: 350.11682

2-(4-Chloro-2-(trifluoromethyl)phenylamino)-5-(2R,3S-O-isopropylidene-4S-O-methyl-tetrahydrofuro-2,3,4-triol-5S)-1,3,4-thiadiazole (**k10**) Yield: 79%. White solid, mp 188.6-189.2°C.^1^H-NMR (CDCl_3_):*δ* 7.98 (d, *J* = 8.8 Hz, 1H, ArH), 7.62 (d, *J* = 2.3 Hz, 1H, ArH), 7.53 (dd, *J* = 8.7, 2.3 Hz, 1H, ArH), 6.21 (s, 1H, NH), 5.99 (d, *J* = 3.6 Hz, 1H, H-1), 5.57 (d, *J* = 3.1 Hz, 1H, H-4), 4.69 (d, *J* = 3.6 Hz, 1H, H-2), 3.98 (d, *J* = 3.1 Hz, 1H, H-3), 3.32 (s, 3H, OCH_3_), 1.55, 1.34 (2s, 6H, Me_2_C). ESI-MS m/z for C_17_H_17_ClF_3_N_3_O_4_S [M+H]+ Found: 452.0. HRMS calcd. for C_17_H_17_ClF_3_N_3_O_4_S [M+H]+ 452.06532. Found: 452.06454

2-(2,5-(ditrifluoromethyl)phenylamino)-5-(2R,3S-O-isopropylidene-4S-O-methyl-tetrahydrofuro-2,3,4-triol-5S)-1,3,4-thiadiazole (**k11**) Yield: 87%. White solid, mp 120.1-121.0°C.^1^H-NMR (CDCl_3_):*δ* 8.44 (s, 1H, ArH), 7.77 (m, 1H, ArH), 7.52 (br-s, 1H, NH), 7.43 (m, 1H, ArH), 6.00 (d, *J* = 3.6 Hz, 1H, H-1), 5.60 (d, *J* = 3.2 Hz, 1H, H-4), 4.71 (d, *J* = 3.6 Hz, 1H, H-2), 4.00 (d, *J* = 3.2 Hz, 1H, H-3), 3.34 (s, 3H, OCH_3_), 1.56, 1.37 (2s, 6H, Me_2_C). ESI-MS m/z for C_18_H_17_F_6_N_3_O_4_S [M+Na]+ Found: 508.1. HRMS calcd. for C_18_H_17_F_6_N_3_O_4_S [M+H]+ 486.09167. Found: 486.09021

2-(4-Bromophenylamino)-5-(2R,3S-O-isopropylidene-4S-O-ethyl-tetrahydrofuro-2,3,4-triol-5S)-1,3,4-thiadiazol (**l1**) Yield: 86%. White solid, mp 213.5-214°C.^1^H-NMR (DMSO-d6):*δ* 10.52 (s, 1H, NH), 7.63–7.60 (m, 2H, ArH), 7.54–7.50 (m, 2H, ArH), 5.98 (d, *J* = 3.7 Hz, 1H, H-1), 5.35 (d, *J* = 3.0 Hz, 1H, H-4), 4.78 (d, *J* = 3.7 Hz, 1H, H-2), 4.06 (d, *J* = 3.0 Hz, 1H, H-3), 3.65, 3.36 (2m, 2H, CH_3_CH_2_), 1.48, 1.31 (2s, 6H, Me_2_C), 1.11 (t, *J* = 6.9 Hz, 3H, CH_3_CH_2_). ESI-MS m/z for C_17_H_20_BrN_3_O_4_S [M+Na]+ Found: 464.0. HRMS calcd. for C_17_H_20_BrN_3_O_4_S [M+H]+ 442.04307. Found: 442.04236

2-(4-Tolylamino)-5-(2R,3S-O-isopropylidene-4S-O-ethyl-tetrahydrofuro-2,3,4-triol-5S)-1,3,4-thiadiazole (**l2**) Yield: 82%. White solid, mp 189.4-193.7°C.^1^H-NMR (CDCl_3_):*δ* 9.91 (s, 1H, NH), 7.32–7.29 (m, 2H, ArH), 7.17 (m, 2H, ArH), 6.01 (d, *J* = 3.6 Hz, 1H, H-1), 5.60 (d, *J* = 3.1 Hz, 1H, H-4), 4.66 (d, *J* = 3.7 Hz, 1H, H-2), 4.05 (d, *J* = 3.1 Hz, 1H, H-3), 3.59, 3.34 (2m, 2H, CH_3_CH_2_), 2.33 (s, 3H, Ar-CH_3_), 1.57, 1.36 (2s, 6H, Me_2_C), 1.10 (t, *J* = 7.0 Hz, 3H, CH_3_CH_2_). ESI-MS m/z for C_18_H_23_N_3_O_4_S [M+H]+ Found: 378.1. HRMS calcd. for C_18_H_23_N_3_O_4_S [M+H]+ 378.14820. Found: 378.14789

2-(4-Methoxyphenylamino)-5-(2R,3S-O-isopropylidene-4S-O-ethyl-tetrahydrofuro-2,3,4-triol-5S)-1,3,4-thiadiazole (**l3**) Yield: 68%. Pale yellow solid, mp 163.1-165.4°C.^1^H-NMR (CDCl_3_):*δ* 9.81 (s, 1H, NH), 7.36–7.32 (m, 2H, ArH), 6.94-6.90 (m, 2H, ArH), 6.00 (d, *J* = 3.6 Hz, 1H, H-1), 5.58 (d, *J* = 3.1 Hz, 1H, H-4), 4.65 (d, *J* = 3.7 Hz, 1H, H-2), 4.03 (d, *J* = 3.0 Hz, 1H, H-3), 3.81 (s, 3H, CH_3_O), 3.57, 3.35 (2m, 2H, CH_3_CH_2_), 1.56, 1.35 (2s, 6H, Me_2_C), 1.10 (t, *J* = 7.0 Hz, 3H, CH_3_CH_2_). ESI-MS m/z for C_18_H_23_N_3_O_5_S [M+H]+ Found: 394.1. HRMS calcd. for C_18_H_23_N_3_O_5_S [M+H]+ 394.14312. Found: 394.14233

2-(2,4-Dimethylphenylamino)-5-(2R,3S-O-isopropylidene-4S-O-ethyl-tetrahydrofuro-2,3,4-triol-5S)-1,3,4-thiadiazole (**l4**) Yield: 77%. White solid, mp 134.7-135.5°C.^1^H-NMR (CDCl_3_):*δ* 8.21 (s, 1H, NH), 7.32 (d, *J* = 8.0 Hz, 1H, ArH), 7.06–7.00 (m, 2H, ArH), 5.96 (d, *J* = 3.6 Hz, 1H, H-1), 5.53 (d, *J* = 3.1 Hz, 1H, H-4), 4.63 (d, *J* = 3.7 Hz, 1H, H-2), 4.01 (d, *J* = 3.1 Hz, 1H, H-3), 3.55, 3.33 (2m, 2H, CH_3_CH_2_), 2.32 (s, 6H, Ar-CH_3_), 1.54, 1.34 (2s, 6H, Me_2_C), 1.08 (t, *J* = 7.0 Hz, 3H, CH_3_CH_2_). ESI-MS m/z for C_19_H_25_N_3_O_4_S [M+Na]+ Found: 414.1. HRMS calcd. for C_19_H_25_N_3_O_4_S [M+H]+ 392.16385. Found: 392.16321

2-(3,4-Dichlorophenylamino)-5-(2R,3S-O-isopropylidene-4S-O-ethyl-tetrahydrofuro-2,3,4-triol-5S)-1,3,4-thiadiazole (**l5**) Yield: 80%. White solid, mp 191.5-191.7°C.^1^H-NMR (CDCl_3_):*δ* 10.25 (s, 1H, NH), 7.60 (d, *J* = 2.6 Hz, 1H, ArH), 7.46–7.30 (m, 2H, ArH), 6.04 (d, *J* = 3.6 Hz, 1H, H-1), 5.61 (d, *J* = 3.1 Hz, 1H, H-4), 4.69 (d, *J* = 3.6 Hz, 1H, H-2), 4.08 (d, *J* = 3.1 Hz, 1H, H-3), 3.62, 3.39 (2m, 2H, CH_3_CH_2_), 1.58, 1.37 (2s, 6H, Me_2_C), 1.13 (t, *J* = 7.0 Hz, 3H, CH_3_CH_2_). ESI-MS m/z for C_17_H_19_Cl_2_N_3_O_4_S [M+H]+ Found: 432.0. HRMS calcd. for C_17_H_19_Cl_2_N_3_O_4_S [M+H]+ 432.05461. Found: 432.05469

2-(2,5-Dichlorophenylamino)-5-(2R,3S-O-isopropylidene-4S-O-ethyl-tetrahydrofuro-2,3,4-triol-5S)-1,3,4-thiadiazole (**l6**) Yield: 82%. White solid, mp 124.9-125.3°C.^1^H-NMR (CDCl_3_):*δ* 8.23 (d, *J* = 2.3 Hz, 1H, ArH), 7.69 (s, 1H, NH), 7.30 (m, 1H, ArH),6.98 (dd, *J* = 8.5, 2.4 Hz, 1H, ArH), 6.04 (d, *J* = 3.6 Hz, 1H, H-1), 5.61 (d, *J* = 3.1 Hz, 1H, H-4), 4.69 (d, *J* = 3.6 Hz, 1H, H-2), 4.09 (d, *J* = 3.1 Hz, 1H, H-3), 3.65-3.32 (2 m, 2 H, CH_3_CH_2_), 1.57, 1.37 (2 s, 6 H, Me_2_C), 1.12 (t, *J* = 7.0 Hz, 3H, CH_3_CH_2_). ESI-MS m/z for C_17_H_19_Cl_2_N_3_O_4_S [M+H]+ Found: 432.0. HRMS calcd. for C_17_H_19_Cl_2_N_3_O_4_S [M+H]+ 432.05461. Found: 432.05414

2-(Naphthalen-1-ylamino)-5-(2R,3S-O-isopropylidene-4S-O-ethyl-tetrahydrofuro-2,3,4-triol-5S)-1,3,4-thiadiazole (**l7**) Yield: 74%. Pale yellow solid, mp 53.9-55.5°C.^1^H-NMR (DMSO-d6):*δ* 10.26 (br-s, 1H, NH), 8.23 (m, 1H, ArH), 8.12 (d, *J* = 7.1 Hz, 1H, ArH), 7.96 (m, 1H, ArH), 7.70 (d, *J* = 8.2 Hz, 1H, ArH), 7.60–7.50 (m, 3H, ArH), 5.96 (d, *J* = 3.7 Hz, 1H, H-1), 5.34 (d, *J* = 3.0 Hz, 1H, H-4), 4.77 (d, *J* = 3.7 Hz, 1H, H-2), 4.06 (d, *J* = 3.0 Hz, 1H, H-3), 3.64, 3.36 (2m, 2H, CH_3_CH_2_), 1.48, 1.30 (2s, 6H, Me_2_C), 1.06 (t, *J* = 7.0 Hz, 3H, CH_3_CH_2_). ESI-MS m/z for C_21_H_23_N_3_O_4_S [M-H] Found: 412.0. HRMS calcd. for C_21_H_23_N_3_O_4_S [M+H]+ 414.14820. Found: 414.14752

2-(4-Chloro-3-(trifluoromethyl)phenylamino)-5-(2R,3S-O-isopropylidene-4S-O-ethyl-tetrahydrofuro-2,3,4-triol-5S)-1,3,4-thiadiazole (**l8**) Yield: 90%. White solid, mp 153.8-154.3°C.^1^H-NMR (CDCl_3_):*δ* 10.65 (br-s, 1H, NH), 7.84 (d, *J* = 2.6 Hz, 1H, ArH), 7.62 (dd, *J* = 8.7, 2.6 Hz, 1H, ArH), 7.50 (m, 1H, ArH), 6.04 (d, *J* = 3.6 Hz, 1H, H-1), 5.61 (d, *J* = 3.1 Hz, 1H, H-4), 4.70 (d, *J* = 3.6 Hz, 1H, H-2), 4.08 (d, *J* = 3.1 Hz, 1H, H-3), 3.64, 3.39 (2m, 2H, CH_3_CH_2_), 1.59, 1.38 (2s, 6H, Me_2_C), 1.13 (t, *J* = 7.0 Hz, 3H, CH_3_CH_2_). ESI-MS m/z for C_18_H_19_ClF_3_N_3_O_4_S [M-H] Found: 464.0. HRMS calcd. for C_18_H_19_ClF_3_N_3_O_4_S [M+H]+ 466.08097. Found: 466.08093

2-(Phenylamino)-5-(2R,3S-O-isopropylidene-4S-O-allyl-tetrahydrofuro-2,3,4-triol-5S)-1,3,4-thiadiazole (**l9**) Yield: 88%. White solid, mp 176.1-177.5°C.^1^H-NMR (CDCl_3_):*δ* 10.41 (s, 1H, NH), 7.41 (m, 4H, ArH), 7.09 (t, *J* = 7.1 Hz, 1H, ArH), 6.03 (d, *J* = 3.6 Hz, 1H, H-1), 5.64 (d, *J* = 3.1 Hz, 1H, H-4), 4.68 (d, *J* = 3.6 Hz, 1H, H-2), 4.07 (d, *J* = 3.0 Hz, 1H, H-3), 3.60, 3.35 (2m, 2H, CH_3_CH_2_), 1.58, 1.37 (2s, 6H, Me_2_C), 1.11 (t, *J* = 7.0 Hz, 3H, CH_3_CH_2_). ESI-MS m/z for C_17_H_21_N_3_O_4_S [M+H]+ Found: 364.1. HRMS calcd. for C_17_H_21_N_3_O_4_S [M+H]+ 364.13255. Found: 364.13220

2-(4-Chloro-2-(trifluoromethyl)phenylamino)-5-(2R,3S-O-isopropylidene-4S-O-ethyl-tetrahydrofuro-2,3,4-triol-5S)-1,3,4-thiadiazole (**l10**) Yield: 86%. White solid, mp 65.7-67.0°C.^1^H-NMR (CDCl_3_):*δ* 7.95 (d, *J* = 8.8 Hz, 1H, ArH), 7.63 (d, *J* = 2.4 Hz, 1H, ArH), 7.53 (dd, *J* = 8.6, 2.3 Hz, 1H, ArH), 5.99 (d, *J* = 3.6 Hz, 1H, H-1), 5.81 (s, 1H, NH), 5.57 (d, *J* = 3.1 Hz, 1H, H-4), 4.66 (d, *J* = 3.6 Hz, 1H, H-2), 4.06 (d, *J* = 3.1 Hz, 1H, H-3), 3.59, 3.36 (2m, 2H, CH_3_CH_2_), 1.55, 1.35 (2s, 6H, Me_2_C), 1.09 (t, *J* = 7.0 Hz, 3H, CH_3_CH_2_). ESI-MS m/z for C_18_H_19_ClF_3_N_3_O_4_S [M+Na]+ Found: 488.0. HRMS calcd. for C_18_H_19_ClF_3_N_3_O_4_S [M+H]+ 466.08097. Found: 466.07993

2-(2,5-(ditrifluoromethyl)phenylamino)-5-(2R,3S-O-isopropylidene-4S-O-ethyl-tetrahydrofuro-2,3,4-triol-5S)-1,3,4-thiadiazole (**l11**) Yield: 84%. White solid, mp 121.2-122.4°C.^1^H-NMR (CDCl_3_):*δ* 8.36 (s, 1H, NH), 7.78 (d, *J* = 8.0 Hz, 2H, ArH), 7.44 (d, *J* = 8.2 Hz, 1H, ArH), 6.02 (d, *J* = 3.6 Hz, 1H, H-1), 5.59 (d, *J* = 3.1 Hz, 1H, H-4), 4.68 (d, *J* = 3.6 Hz, 1H, H-2), 4.08 (d, *J* = 3.1 Hz, 1H, H-3), 3.62, 3.38 (2m, 2H, CH_3_CH_2_), 1.56, 1.37 (2s, 6H, Me_2_C), 1.12 (t, *J* = 7.0 Hz, 3H, CH_3_CH_2_). ESI-MS m/z for C_19_H_19_F_6_N_3_O_4_S [M+H]+ Found: 500.1. HRMS calcd. for C_19_H_19_F_6_N_3_O_4_S [M+H]+ 500.10732. Found: 500.10635

### Fungicidal assays

Each of the test compounds were dissolved in DMSO. Fungicidal activities of compounds **k**, and **l** against Sclerotinia sclerotiorum, P. CapasiciLeonian, Botrytis cinerea, Rhizoctonia solani, Pyricularia oryae and Phomopsis asparagi were evaluated using the mycelium growth rate test.

Inhibition rates of compounds **k** and **l** against Sclerotinia sclerotiorum, P. CapasiciLeonian, Botrytis cinerea, Rhizoctonia solani, Pyricularia oryae and Phomopsis asparagi at 50 μg/mL were determined first and the results are shown in SI. Then EC_50_ values were estimated using logit analysis. The commercial fungicide chlorothalonil was used as a control in the above bioassay.

### CoMFA and CoMSIA model

All computational studies were performed using SYBYL-X2.0 software. The compounds were built from fragments in the SYBYL database. Each structure was fully geometry-optimized by MINIMIZE module using the standard MMFF94 force field with a distance-dependent dielectric function and a 0.21 kJ/mol•nm energy gradient convergence criterion 1000 times. After optimization, considering all the carbon, nitrogen, sulfur atoms and oxygen atoms, superimposition of the molecules was carried out by Alignment Database module, using the most active compound **k8** as a template molecule for aligning the other analogues.

For each of the alignments, calculation of CoMFA steric and electrostatic fields were separately carried out at each lattice intersection on a regularly spaced grid of 1 nm x 1 nm x 1 nm units in X, Y, and Z directions. The van der Waals potential and columbic terms, which represent the steric and electrostatic terms, respectively, were calculated using the standard Tripos force field. A distance dependent dielectric constant of 1.00 was used. An sp3 carbon atom with a van der Waals radius of 1.52 Å and +1.0 charge was selected as the probe to compute the steric and electrostatic fields. Values of the steric and electrostatic energy were truncated at 30 kcal/mol. The electrostatic contributions were ignored at the lattice intersection with maximal steric interactions.

CoMSIA calculates similarity indices at the intersections of a surrounding lattice. The similarity indices descriptors were derived with the same lattice box used in CoMFA. The five CoMSIA fields available within SYBYL (steric, electrostatic, hydrophobic, hydrogen bond donor and acceptor) were calculated at the grid lattice point using a probe atom of 1 Å radius as well as the charge, hydrophobic and hydrogen bond properties of H.

## Conclusion

In this study, twenty two xylofuranose modified 1,3,4-thiadiazole derivatives were designed and synthesized. Some of the title compounds exhibited excellent antifungal activities against Sclerotinia sclerotiorum, among which, compounds **k1**, **k8**, **l1** and **l5** showed even better fungicidal activities than the commercial fungicide Chlorothalonil. Based on the COMFA and CoMSIA models, we provided a way to enhance the antifungal activity by changing the hydrophilicity, electrostatic property and volume of the substituents. Our suggested requirements of the molecular structures identified through 3D-QSAR are consistent with the experimental results, which can help in designing more active fungicides.

## Supporting information

S1 Table Fungicidal activity of target compounds against six fungus species. (DOCX)

S2 Table HRMS spectral data of the target compounds. (DOCX)

S3 Table Predictive toxicity and log P values of the target compounds. (DOCX)

S4 Table The target name, the PDB ID and feature number of 22 compounds. (DOCX)

S1 File NMR and HRMS spectra of the target compounds. (DOC)

## References

[1] Clerici F, Pocar D, Guido M, Loche A, Perlini V, Brufani M. Synthesis of 2-Amino-5-sulfanyl-1,3,4-thiadiazole Derivatives and Evaluation of Their Antidepressant and Anxiolytic Activity. Journal of Medicinal Chemistry. 2001;44(6):931–936. 10.1021/jm001027w 11300875

[2] Oruç EE, Rollas S, Kandemirli F, Shvets N, Dimoglo AS. 1,3,4-Thiadiazole Derivatives. Synthesis, Structure Elucidation, and Structure–Antituberculosis Activity Relationship Investigation. Journal of Medicinal Chemistry. 2004;47(27):6760–6767. 10.1021/jm0495632 15615525

[3] Kalidhar U, Kaur A. 1,3,4-Thiadiazole derivatives and their biological activities: a review. Res J Pharm, Biol Chem Sci. 2011;2(4):1091–1106.

[4] Kamal M, Shakya A, Jawaid T. 1,3,4-Thiadiazole as antimicrobial agent: a review. Int J Biomed Res. 2011;2(1):41–61. 10.7439/ijbr.v2i1.80

[5] Jain AK, Sharma S, Vaidya A, Ravichandran V, Agrawal RK. 1,3,4-Thiadiazole and its Derivatives: A Review on Recent Progress in Biological Activities. Chemical Biology & Drug Design. 2013;81(5):557–576. 10.1111/cbdd.12125 23452185

[6] Hu Y, Li CY, Wang XM, Yang YH, Zhu HL. 1,3,4-Thiadiazole: Synthesis, Reactions, and Applications in Medicinal, Agricultural, and Materials Chemistry. Chemical Reviews. 2014;114(10):5572–5610. 10.1021/cr400131u 24716666

[7] CHEN Chuan-Bing ZHANG DMWSWYG Zheng-Wen. Synthesis of N-[[(5-mercapto-1,3,4-thiadiazol-2-yl)amino]carbonyl]benzamide and 2-(phenoxy)-N-[[(5-mercapto-1,3,4-thiadiazol-2-yl)amino]carbonyl]acetamide derivatives and determination of their activity as plant growth regulators. Chinese Journal of Organic Chemistry. 2007;27(11):1444.

[8] Chen CJ, Song BA, Yang S, Xu GF, Bhadury PS, Jin LH, et al Synthesis and antifungal activities of 5-(3,4,5-trimethoxyphenyl)-2-sulfonyl-1,3,4-thiadiazole and 5-(3,4,5-trimethoxyphenyl)-2-sulfonyl-1,3,4-oxadiazole derivatives. Bioorganic & Medicinal Chemistry. 2007;15(12):3981–3989. 10.1016/j.bmc.2007.04.014 17452108

[9] Wang T, Miao W, Wu S, Bing G, Zhang X, Qin Z, et al Synthesis, Crystal Structure, and Herbicidal Activities of 2-Cyanoacrylates Containing 1,3,4-Thiadiazole Moieties. Chinese Journal of Chemistry. 2011;29(5):959–967. 10.1002/cjoc.201190196

[10] Li F, Mo Q, Duan W, Lin G, Cen B, Chen N, et al Synthesis and insecticidal activities of N-(5-dehydroabietyl-1,3,4-thiadiazol-2-yl)-benzenesulfonamides. Medicinal Chemistry Research. 2014;23(10):4420–4426. 10.1007/s00044-014-1009-x

[11] He LE, Wu YY, Zhang HY, Liu MY, Shi DQ. Design, Synthesis, and Herbicidal Evaluation of Novel Uracil Derivatives Containing 1,3,4-Thiadiazolyl Moiety. Journal of Heterocyclic Chemistry. 2015;52(5):1308–1313. 10.1002/jhet.2160

[12] Cui ZN, Li YS, Hu DK, Tian H, Jiang JZ, Wang Y, et al Synthesis and fungicidal activity of novel 2,5-disubstituted-1,3,4-thiadiazole derivatives containing 5-phenyl-2-furan. SCIENTIFIC REPORTS. 2016;6. 10.1038/srep20204 PMC4731749 26822318

[13] Dai H, Li G, Chen J, Shi Y, Ge S, Fan C, et al Synthesis and biological activities of novel 1,3,4-thiadiazole-containing pyrazole oxime derivatives. Bioorganic & Medicinal Chemistry Letters. 2016;26(15):3818–3821. 10.1016/j.bmcl.2016.04.094 27324978

[14] Chen L, Wang DQ, Jin Sh. Synthesis and fungicidal activity of 2-(1,11-undecylidene)-5-substituted imino-?3-1,3,4-thiadiazolines. Chinese Journal of Applied Chemistry. 2002;19(3):212–215.

[15] Chen L, Wang DQ, Jin Sh. Synthesis and fungicidal activity of 2-(1,5-pentamethylene)-5-substituted imino-?3-1,3,4-thiadiazolines. Nongyaoxue Xuebao. 2004;6(1):22–25.

[16] Li JJ, Liang XM, Jin SH, Zhang JJ, Yuan HZ, Qi SH, et al Synthesis, Fungicidal Activity, and Structure–Activity Relationship of Spiro-Compounds Containing Macrolactam (Macrolactone) and Thiadiazoline Rings. Journal of Agricultural and Food Chemistry. 2010;58(5):2659–2663. 10.1021/jf903665f 20041703

[17] Zong G, Zhao H, Jiang R, Zhang J, Liang X, Li B, et al Design, Synthesis and Bioactivity of Novel Glycosylthiadiazole Derivatives. Molecules. 2014;19(6):7832–7849. 10.3390/molecules19067832 24962389 PMC6271630

[18] Copping LG, Duke SO. Natural products that have been used commercially as crop protection agents. Pest Management Science. 2007;63(6):524–554. 10.1002/ps.1378 17487882

[19] McCranie EK, Bachmann BO. Bioactive oligosaccharide natural products. Nat Prod Rep. 2014;31:1026–1042. 10.1039/c3np70128j 24883430 PMC5267508

[20] Huang G, Mei X. Synthetic Glycosylated Natural Products Have Satisfactory Activities. Current Drug Targets. 2014;15(8):780–784. 10.2174/1389450115666140617153348 24942665

[21] Tisler M. Syntheses in the 4-substituted thiosemicarbazide series. Croatica Chemica Acta. 1956;28:147–154.

[22] Krueger EB, Hopkins TP, Keaney MT, Walters MA, Boldi AM. Solution-Phase Library Synthesis of Furanoses. Journal of Combinatorial Chemistry. 2002;4(3):229–238. 10.1021/cc010078r 12005483

[23] Varaprasad CVNS, Barawkar D, Abdellaoui HE, Chakravarty S, Allan M, Chen H, et al Discovery of 3-hydroxy-4-carboxyalkylamidino-5-arylamino-isothiazoles as potent {MEK1} inhibitors. Bioorganic & Medicinal Chemistry Letters. 2006;16(15):3975–3980. 10.1016/j.bmcl.2006.05.019 16725322

[24] Liu X, Ouyang S, Yu B, Liu Y, Huang K, Gong J, et al PharmMapper server: a web server for potential drug target identification using pharmacophore mapping approach. Nucleic Acids Research. 2010;38(suppl_2):W609 10.1093/nar/gkq300 20430828 PMC2896160

[25] Wang X, Pan C, Gong J, Liu X, Li H. Enhancing the Enrichment of Pharmacophore-Based Target Prediction for the Polypharmacological Profiles of Drugs. Journal of Chemical Information and Modeling. 2016;56(6):1175–1183. 10.1021/acs.jcim.5b00690 27187084

