# Supplementary material for: Synthesis, fungicidal evaluation and 3D-QSAR studies of novel 1,3,4-thiadiazole xylofuranose derivatives
Source: PLoS One. 2017 Jul 26;12(7):e0181646. doi: 10.1371/journal.pone.0181646 (PMC5528880; doi:10.1371/journal.pone.0181646)
Supplement: S1 Table — (DOCX) [file pone.0181646.s001.docx]

Table S1. Fungicidal activity of target compounds against six fungus species (% control at 50 µg/mL)

| Compd. | Inhibitory rate/% | | | | | |
| --- | --- | --- | --- | --- | --- | --- |
|  | *S. sclerotiorum* | *P. CapasiciLeonian* | *B. cinerea* | *R. solani* | *P. oryae* | *P. asparagi* |
| **k1** | **98** | 78 | **90** | 81 | **91** | 74 |
| **k2** | 76 | 60 | 75 | 67 | 83 | 60 |
| **k3** | 61 | 27 | 57 | 57 | 62 | 37 |
| **k4** | 86 | 47 | 73 | 54 | 70 | 55 |
| **k5** | **96** | 77 | **90** | 72 | **90** | 57 |
| **k6** | **98** | 88 | **95** | 63 | **92** | **95** |
| **k7** | **95** | 60 | **93** | 74 | 78 | 77 |
| **k8** | **98** | **99** | **94** | 72 | 82 | 73 |
| **k9** | 80 | 3 | 65 | 38 | 58 | 28 |
| **k10** | **96** | 53 | 84 | 43 | 70 | 69 |
| **k11** | 89 | 34 | 49 | 51 | 39 | -16 |
| **l1** | **90** | 62 | 89 | 63 | 84 | 61 |
| **l2** | 87 | 51 | 84 | 71 | 87 | 79 |
| **l3** | 66 | -1 | 46 | 48 | 76 | 75 |
| **l4** | **92** | 23 | 86 | 57 | 83 | 48 |
| **l5** | **97** | **99** | 83 | 69 | **92** | **98** |
| **l6** | **99** | **96** | 88 | 56 | 89 | **98** |
| **l7** | **98** | 63 | 89 | 78 | 88 | **98** |
| **l8** | **97** | **99** | **93** | 75 | 84 | 85 |
| **l9** | 79 | 16 | 40 | 58 | 73 | 86 |
| **l10** | **96** | **96** | 81 | 75 | 80 | 87 |
| **l11** | 73 | 28 | 29 | 37 | 74 | 41 |
| Chlorothalonil | 99 | 95 | 88 | 97 | 87 | 94 |
