# Supplementary material for: Synthesis, fungicidal evaluation and 3D-QSAR studies of novel 1,3,4-thiadiazole xylofuranose derivatives
Source: PLoS One. 2017 Jul 26;12(7):e0181646. doi: 10.1371/journal.pone.0181646 (PMC5528880; doi:10.1371/journal.pone.0181646)
Supplement: S2 Table — (DOCX) [file pone.0181646.s002.docx]

Table S2. HRMS spectral data of the target compounds

| Compd. | molecular formula | detection ion | experimental value | calculated value | deviation (m/z) | deviation (ppm) |
| --- | --- | --- | --- | --- | --- | --- |
| **k1** | C_16_H_18_BrN_3_O_4_S | [M+H]^+^ | 428.02686 | 428.02742 | -0.00056 | -1.30833 |
| **k2** | C_17_H_21_N_3_O_4_S | [M+H]^+^ | 364.13193 | 364.13255 | -0.00062 | -1.70268 |
| **k3** | C_17_H_21_N_3_O_5_S | [M+H]^+^ | 380.12708 | 380.12747 | -0.00039 | -1.02597 |
| **k4** | C_18_H_23_N_3_O_4_S | [M+H]^+^ | 378.14798 | 378.14820 | -0.00022 | -0.58178 |
| **k5** | C_16_H_17_Cl_2_N_3_O_4_S | [M+H]^+^ | 418.03897 | 418.03896 | 0.00001 | 0.02392 |
| **k6** | C_16_H_17_C_l2_N_3_O_4_S | [M+H]^+^ | 418.03854 | 418.03896 | -0.00042 | -1.00469 |
| **k7** | C_20_H_21_N_3_O_4_S | [M+H]^+^ | 400.13208 | 400.13255 | -0.00047 | -1.17461 |
| **k8** | C_17_H_17_ClF_3_N_3_O_4_S | [M+H]^+^ | 452.06512 | 452.06532 | -0.00020 | -0.44241 |
| **k9** | C_16_H_19_N_3_O_4_S | [M+H]^+^ | 350.11682 | 350.11690 | -0.00008 | -0.22850 |
| **k10** | C_17_H_17_ClF_3_N_3_O_4_S | [M+H]^+^ | 452.06454 | 452.06532 | -0.00078 | -1.72541 |
| **k11** | C_18_H_17_F_6_N_3_O_4_S | [M+H]^+^ | 486.09021 | 486.09167 | -0.00146 | -3.00355 |
| **l1** | C_17_H_20_BrN_3_O_4_S | [M+H]^+^ | 442.04236 | 442.04307 | -0.00071 | -1.60618 |
| **l2** | C_18_H_23_N_3_O_4_S | [M+H]^+^ | 378.14789 | 378.14820 | -0.00031 | -0.81978 |
| **l3** | C_18_H_23_N_3_O_5_S | [M+H]^+^ | 394.14233 | 394.14312 | -0.00079 | -2.00435 |
| **l4** | C_19_H_25_N_3_O_4_S | [M+H]^+^ | 392.16321 | 392.16385 | -0.00064 | -1.63197 |
| **l5** | C_17_H_19_C_l2_N_3_O_4_S | [M+H]^+^ | 432.05469 | 432.05461 | 0.00008 | 0.18516 |
| **l6** | C_17_H_19_C_l2_N_3_O_4_S | [M+H]^+^ | 432.05414 | 432.05461 | -0.00047 | -1.08783 |
| **l7** | C_21_H_23_N_3_O_4_S | [M+H]^+^ | 414.14752 | 414.14820 | -0.00068 | -1.64192 |
| **l8** | C_18_H_19_ClF_3_N_3_O_4_S | [M+H]^+^ | 466.08093 | 466.08097 | -0.00004 | -0.08582 |
| **l9** | C_17_H_21_N_3_O_4_S | [M+H]^+^ | 364.13220 | 364.13255 | -0.00035 | -0.96119 |
| **l10** | C_18_H_19_ClF_3_N_3_O_4_S | [M+H]^+^ | 466.07993 | 466.08097 | -0.00104 | -2.23137 |
| **l11** | C_19_H_19_F_6_N_3_O_4_S | [M+H]^+^ | 500.10635 | 500.10732 | -0.00097 | -1.93958 |
