# Supplementary material for: Synthesis, fungicidal evaluation and 3D-QSAR studies of novel 1,3,4-thiadiazole xylofuranose derivatives
Source: PLoS One. 2017 Jul 26;12(7):e0181646. doi: 10.1371/journal.pone.0181646 (PMC5528880; doi:10.1371/journal.pone.0181646)
Supplement: S3 Table — (DOCX) [file pone.0181646.s003.docx]

Table S3. Predictive toxicity and log P values of the target compounds

| Compd. | Log P | Acute toxicity | Carcinogenic toxicity | Mutagenic toxicity |
| --- | --- | --- | --- | --- |
| **k1** | 3.33 | 2.82 | negative | negative |
| **k2** | 2.76 | 3.06 | negative | negative |
| **k3** | 2.31 | 3.09 | negative | negative |
| **k4** | 2.93 | 3.07 | negative | negative |
| **k5** | 3.85 | 3 | negative | negative |
| **k6** | 3.75 | 2.94 | negative | negative |
| **k7** | 3.54 | 3.12 | negative | Partial negative |
| **k8** | 3.9 | 2.99 | negative | negative |
| **k9** | 2.28 | 3.02 | negative | negative |
| **k10** | 3.68 | 2.92 | negative | negative |
| **k11** | 3.97 | 2.87 | negative | negative |
| **l1** | 3.67 | 2.84 | negative | negative |
| **l2** | 3.11 | 3.08 | negative | negative |
| **l3** | 2.65 | 3.11 | negative | negative |
| **l4** | 3.28 | 3.09 | negative | negative |
| **l5** | 4.2 | 3.02 | negative | negative |
| **l6** | 4.1 | 2.96 | negative | negative |
| **l7** | 3.88 | 3.14 | negative | Partial negative |
| **l8** | 4.25 | 3.01 | negative | negative |
| **l9** | 2.63 | 3.04 | negative | negative |
| **l10** | 4.03 | 2.94 | negative | negative |
| **l11** | 4.32 | 2.89 | negative | negative |
