# Supplementary material for: Synthesis, fungicidal evaluation and 3D-QSAR studies of novel 1,3,4-thiadiazole xylofuranose derivatives
Source: PLoS One. 2017 Jul 26;12(7):e0181646. doi: 10.1371/journal.pone.0181646 (PMC5528880; doi:10.1371/journal.pone.0181646)
Supplement: S4 Table — (DOCX) [file pone.0181646.s004.docx]

Table S4. The target name, the PDB ID and feature number of 22 compounds

| Compd. | PDB ID | Target Name | Number of Feature |
| --- | --- | --- | --- |
| **k1** | 3EML | Adenosine receptor A2a | 9 |
| **k2** | 3DP2 | NONE | 9 |
| **k3** | 3DP2 | NONE | 9 |
| **k4** | 1UT6 | Acetylcholinesterase | 9 |
| **k5** | 1HE2 | Flavin reductase | 9 |
| **k6** | 2H7L | Enoyl-[acyl-carrier-protein] reductase [NADH] | 9 |
| **k7** | 1UT6 | Acetylcholinesterase | 9 |
| **k8** | 2ITP | Epidermal growth factor receptor | 9 |
| **k9** | 3DP2 | NONE | 9 |
| **k10** | 1UT6 | Acetylcholinesterase | 9 |
| **k11** | 2ITP | Epidermal growth factor receptor | 9 |
| **l1** | 1UT6 | Acetylcholinesterase | 9 |
| **l2** | 3DP2 | NONE | 9 |
| **l3** | 1HE2 | Flavin reductase | 9 |
| **l4** | 1DTL | Troponin C, slow skeletal and cardiac muscles | 9 |
| **l5** | 2H7L | Enoyl-[acyl-carrier-protein] reductase [NADH] | 9 |
| **l6** | 1UT6 | Acetylcholinesterase | 9 |
| **l7** | 1HE2 | Flavin reductase | 9 |
| **l8** | 1HE2 | Flavin reductase | 9 |
| **l9** | 1HE2 | Flavin reductase | 9 |
| **l10** | 1DTL | Troponin C, slow skeletal and cardiac muscles | 9 |
| **l11** | 1UT6 | Acetylcholinesterase | 9 |
