# Supplementary material for: Synthesis, fungicidal evaluation and 3D-QSAR studies of novel 1,3,4-thiadiazole xylofuranose derivatives
Source: PLoS One. 2017 Jul 26;12(7):e0181646. doi: 10.1371/journal.pone.0181646 (PMC5528880; doi:10.1371/journal.pone.0181646)
Supplement: S1 File — (DOC) [file pone.0181646.s005.doc]

Synthesis, fungicidal evaluation and 3D-QSAR studies of 1,3,4-thiadiazole xylofuranose derivatives

Guanghui Zong^1^, Xiaojing Yan^2^, Jiawei Bi^1^, Rui Jiang^1^, Yinan Qin^1^, Huizhu Yuan^2^, Huizhe Lu^1,*^, Yanhong Dong^1^, Shuhui Jin^1^, Jianjun Zhang^1,*^

(^1^ Key Laboratory of Pesticide Chemistry and Application Technology, College of Science, China Agricultural University, Beijing 100193)

(^2^ The Institute of Plant Protection, Chinese Academy of Agricultural Sciences, Beijing 100193)

| **Supporting Information** | | |
| --- | --- | --- |
| NMR and HRMS spectra of the target compounds | | **Page No.** |
| **1** | ^1^H NMR spectrum of compound **k1** | S-4 |
| **2** | HRMS spectrum of compound **k1** | S-4 |
| **3** | ^1^H NMR spectrum of compound **k2** | S-5 |
| **4** | HRMS spectrum of compound **k2** | S-5 |
| **5** | ^1^H NMR spectrum of compound **k3** | S-6 |
| **6** | HRMS spectrum of compound **k3** | S-6 |
| **7** | ^1^H NMR spectrum of compound **k4** | S-7 |
| **8** | HRMS spectrum of compound **k4** | S-7 |
| **9** | ^1^H NMR spectrum of compound **k5** | S-8 |
| **10** | HRMS spectrum of compound **k5** | S-8 |
| **11** | ^1^H NMR spectrum of compound **k6** | S-9 |
| **12** | HRMS spectrum of compound **k6** | S-9 |
| **13** | ^1^H NMR spectrum of compound **k7** | S-10 |
| **14** | HRMS spectrum of compound **k7** | S-10 |
| **15** | ^1^H NMR spectrum of compound **k8** | S-11 |
| **16** | HRMS spectrum of compound **k8** | S-11 |
| **17** | ^1^H NMR spectrum of compound **k9** | S-12 |
| **18** | HRMS spectrum of compound **k9** | S-12 |
| **19** | ^1^H NMR spectrum of compound **k10** | S-13 |
| **20** | HRMS spectrum of compound **k10** | S-13 |
| **21** | ^1^H NMR spectrum of compound **k11** | S-14 |
| **22** | HRMS spectrum of compound **k11** | S-14 |
| **23** | ^1^H NMR spectrum of compound **l1** | S-15 |
| **24** | HRMS spectrum of compound **l1** | S-15 |
| **25** | ^1^H NMR spectrum of compound **l2** | S-16 |
| **26** | HRMS spectrum of compound **l2** | S-16 |
| **27** | ^1^H NMR spectrum of compound **l3** | S-17 |
| **28** | HRMS spectrum of compound **l3** | S-17 |
| **29** | ^1^H NMR spectrum of compound **l4** | S-18 |
| **30** | HRMS spectrum of compound **l4** | S-18 |
| **31** | ^1^H NMR spectrum of compound **l5** | S-19 |
| **32** | HRMS spectrum of compound **l5** | S-19 |
| **33** | ^1^H NMR spectrum of compound **l6** | S-20 |
| **34** | HRMS spectrum of compound **l6** | S-20 |
| **35** | ^1^H NMR spectrum of compound **l7** | S-21 |
| **36** | HRMS NMR spectrum of compound **l7** | S-21 |
| **37** | ^1^H NMR spectrum of compound **l8** | S-22 |
| **38** | ^13^C NMR spectrum of compound **l8** | S-22 |
| **39** | HSQC NMR spectrum of compound **l8** | S-23 |
| **40** | COSYNMR spectrum of compound **l8** | S-23 |
| **41** | HMBC NMR spectrum of compound **l8** | S-24 |
| **42** | HRMS spectrum of compound **l8** | S-24 |
| **43** | ^1^H NMR spectrum of compound **l9** | S-25 |
| **44** | HRMS spectrum of compound **l9** | S-25 |
| **45** | ^1^H NMR spectrum of compound **l10** | S-26 |
| **46** | HRMS spectrum of compound **l10** | S-26 |
| **47** | ^1^H NMR spectrum of compound **l11** | S-27 |
| **48** | HRMS spectrum of compound **l11** | S-27 |

^1^H NMR spectrum of compound **k1**

HRMS spectrum of compound **k1**

^1^H NMR spectrum of compound **k2**

HRMS spectrum of compound **k2**

^1^H NMR spectrum of compound **k3**

HRMS spectrum of compound **k3**

^1^H NMR spectrum of compound **k4**

HRMS spectrum of compound **k4**

^1^H NMR spectrum of compound **k5**

HRMS spectrum of compound **k5**

^1^H NMR spectrum of compound **k6**

HRMS spectrum of compound **k6**

^1^H NMR spectrum of compound **k7**

HRMS spectrum of compound **k7**

^1^H NMR spectrum of compound **k8**

HRMS spectrum of compound **k8**

^1^H NMR spectrum of compound **k9**

HRMS spectrum of compound **k9**

^1^H NMR spectrum of compound **k10**

HRMS spectrum of compound **k10**

^1^H NMR spectrum of compound **k11**

HRMS spectrum of compound **k11**

^1^H NMR spectrum of compound **l1**

HRMS spectrum of compound **l1**

^1^H NMR spectrum of compound **l2**

HRMS spectrum of compound **l2**

^1^H NMR spectrum of compound **l3**

HRMS spectrum of compound **l3**

^1^H NMR spectrum of compound **l4**

HRMS spectrum of compound **l4**

^1^H NMR spectrum of compound **l5**

HRMS spectrum of compound **l5**

^1^H NMR spectrum of compound **l6**

HRMS spectrum of compound **l6**

^1^H NMR spectrum of compound **l7**

HRMS spectrum of compound **l7**

^1^H NMR spectrum of compound **l8**

^13^C NMR spectrum of compound **l8**


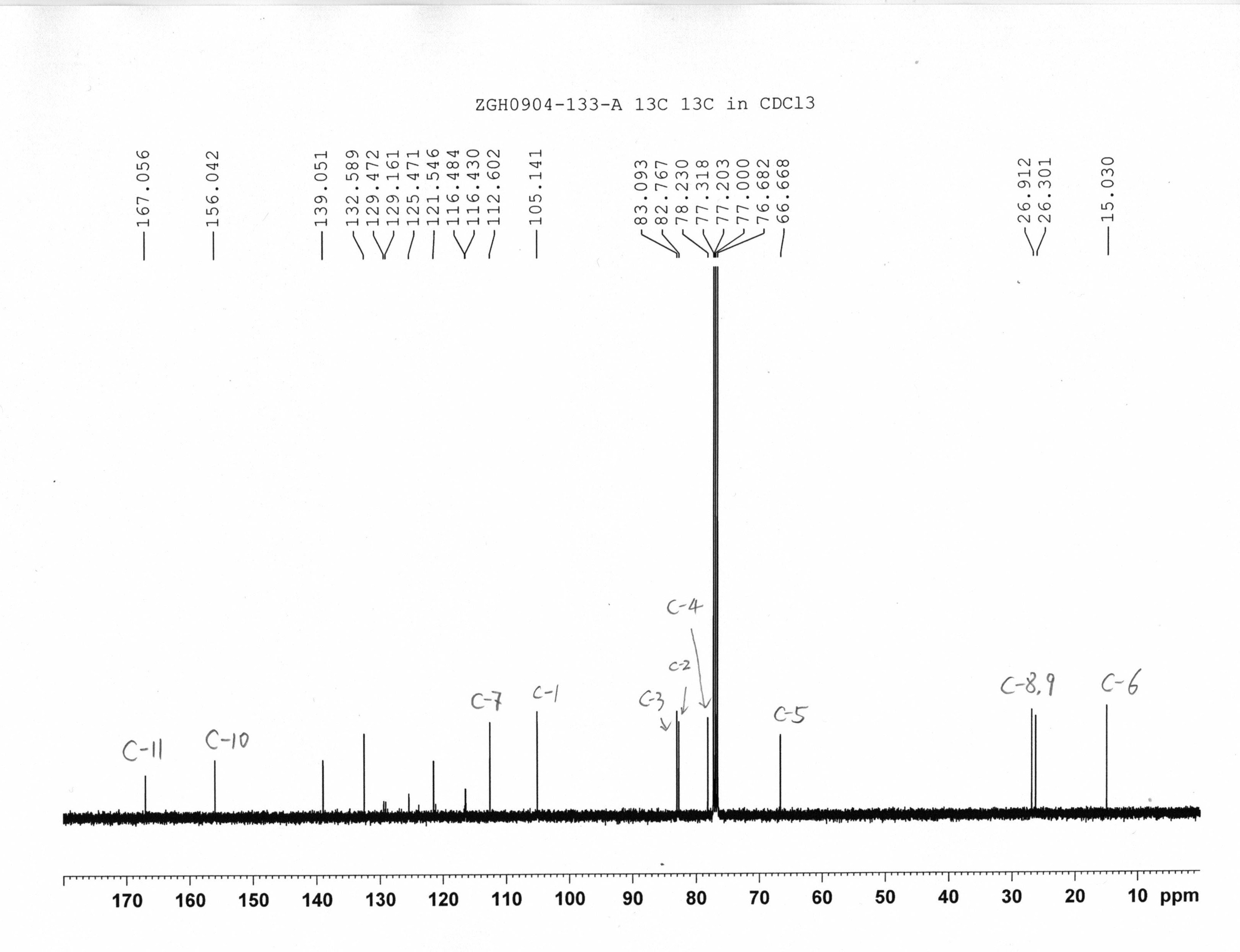


HSQC NMR spectrum of compound **l8**


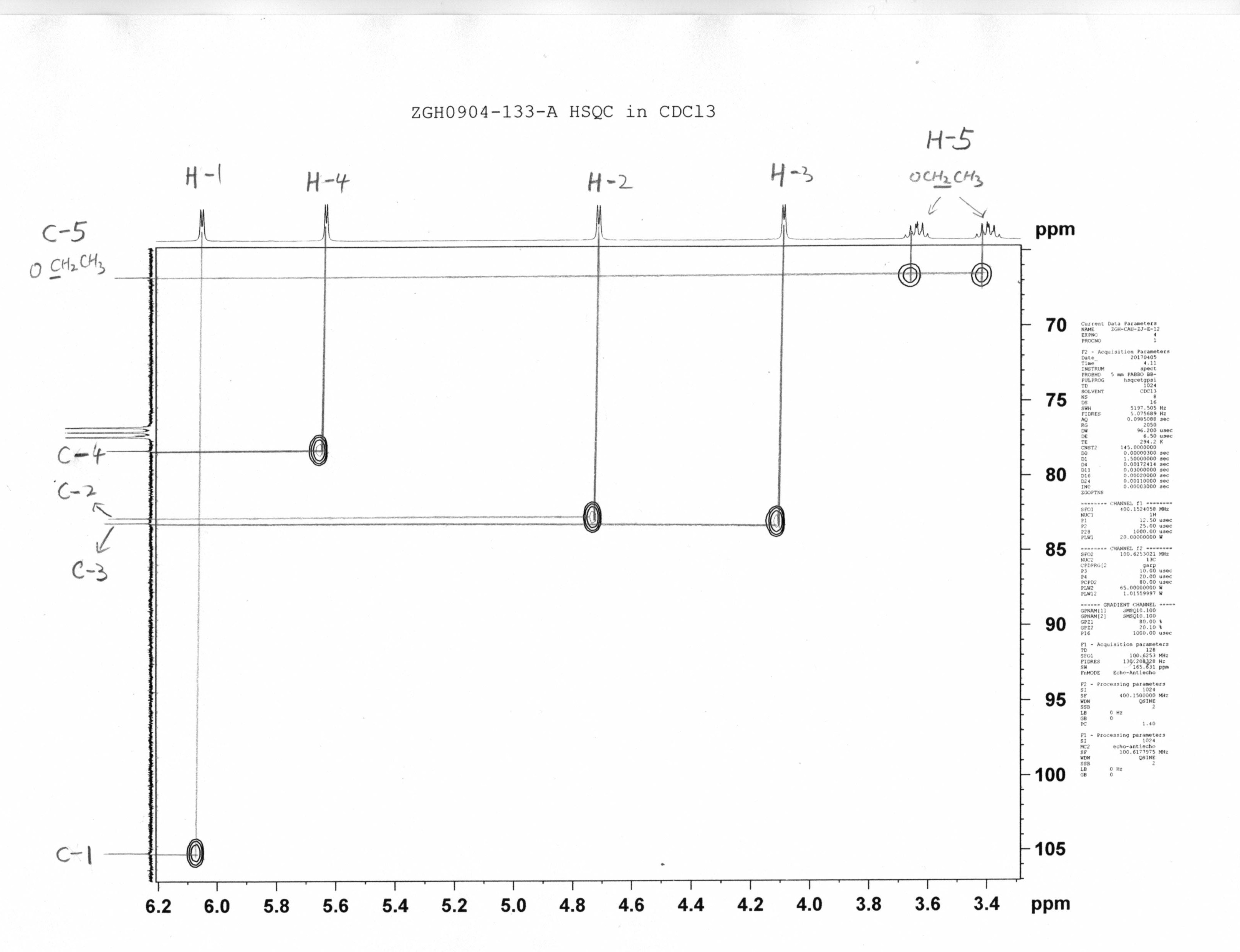


COSY NMR spectrum of compound **l8**


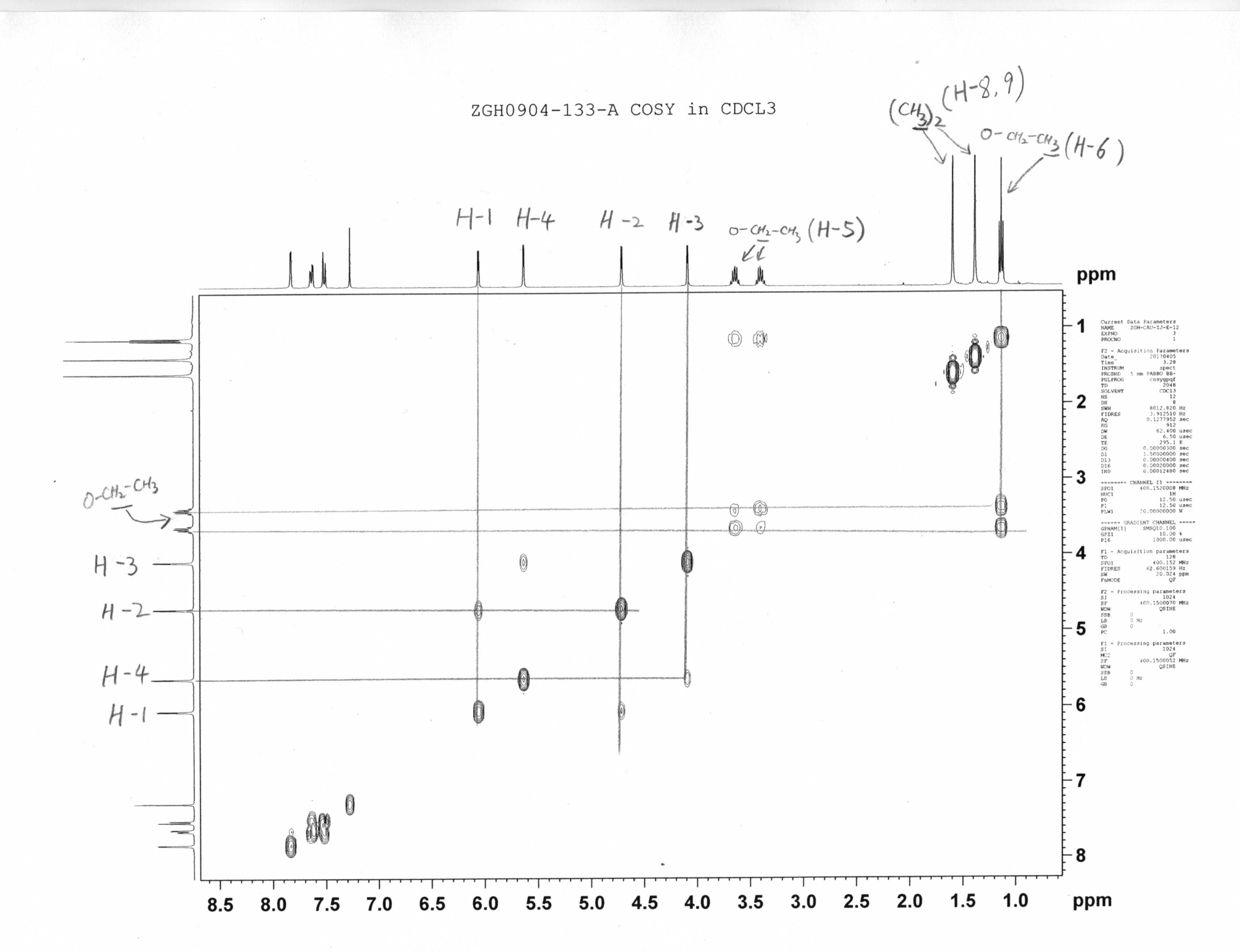


HMBC NMR spectrum of compound **l8**


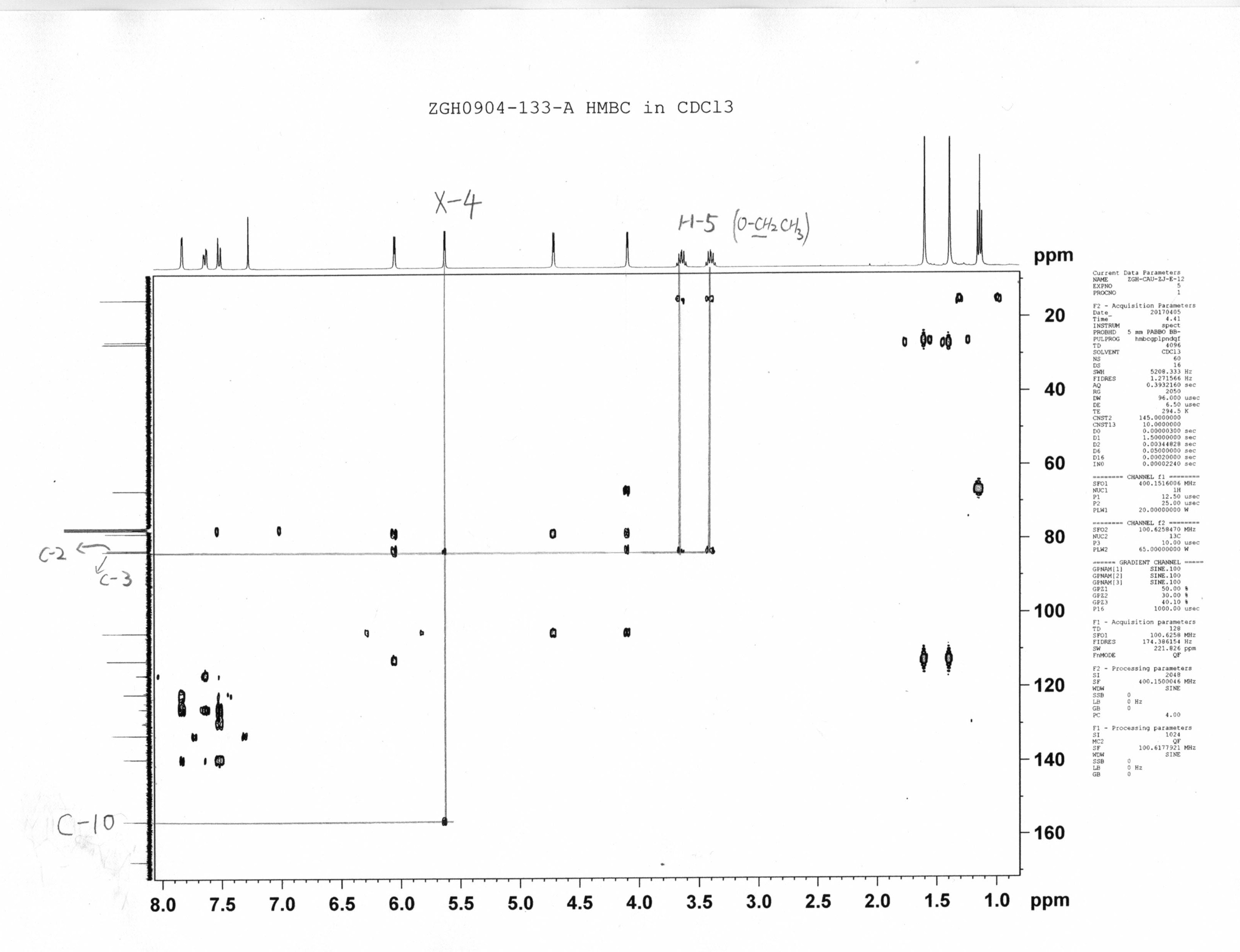


HRMS spectrum of compound **l8**

^1^H NMR spectrum of compound **l9**

HRMS spectrum of compound **l9**

^1^H NMR spectrum of compound **l10**

HRMS spectrum of compound **l10**

^1^H NMR spectrum of compound **l11**

HRMS spectrum of compound **l11**
